# Laser solid-phase synthesis of single-atom catalysts

**DOI:** 10.1038/s41377-021-00603-9

**Published:** 2021-08-18

**Authors:** Yudong Peng, Jianyun Cao, Yang Sha, Wenji Yang, Lin Li, Zhu Liu

**Affiliations:** 1 Department of Materials, School of Natural Sciences, The University of Manchester, Manchester, M13 9PL UK; 2 National Graphene Institute, The University of Manchester, Manchester, M13 9PL UK; 3 Laser Processing Research Centre, Department of Mechanical, Aerospace and Civil Engineering, The University of Manchester, Manchester, M13 9PL UK

**Keywords:** Solid-state lasers, Photonic devices

## Abstract

Single-atom catalysts (SACs) with atomically dispersed catalytic sites have shown outstanding catalytic performance in a variety of reactions. However, the development of facile and high-yield techniques for the fabrication of SACs remains challenging. In this paper, we report a laser-induced solid-phase strategy for the synthesis of Pt SACs on graphene support. Simply by rapid laser scanning/irradiation of a freeze-dried electrochemical graphene oxide (EGO) film loaded with chloroplatinic acid (H_2_PtCl_6_), we enabled simultaneous pyrolysis of H_2_PtCl_6_ into SACs and reduction/graphitization of EGO into graphene. The rapid freezing of EGO hydrogel film infused with H_2_PtCl_6_ solution in liquid nitrogen and the subsequent ice sublimation by freeze-drying were essential to achieve the atomically dispersed Pt. Nanosecond pulsed infrared (IR; 1064 nm) and picosecond pulsed ultraviolet (UV; 355 nm) lasers were used to investigate the effects of laser wavelength and pulse duration on the SACs formation mechanism. The atomically dispersed Pt on graphene support exhibited a small overpotential of −42.3 mV at −10 mA cm^−2^ for hydrogen evolution reaction and a mass activity tenfold higher than that of the commercial Pt/C catalyst. This method is simple, fast and potentially versatile, and scalable for the mass production of SACs.

## Abstract (web summary)

Synthesis of Pt single atom catalysts is achieved by one-step laser irradiation of “isolated” H_2_PtCl_6_ precursor on graphene oxide, via filtration and freeze-drying sublimation, by simultaneous pyrolysis of the precursor and reduction of graphene oxide.

## Graphical abstract

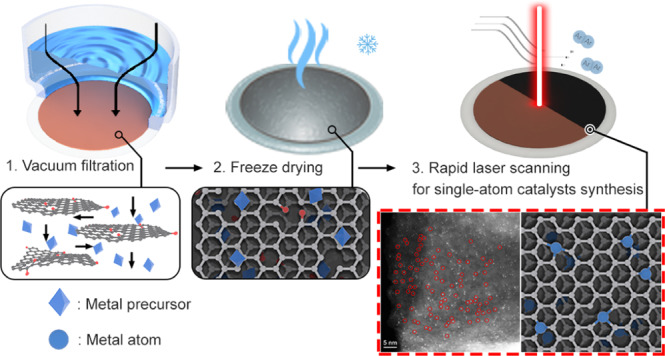

## Introduction

Single-atom catalysts (SACs) are a class of catalysts in which individual and isolated metal atoms are anchored to supports. Due to their maximized atom utilization and unique coordination environments, SACs have emerged as a new frontier in heterogeneous catalysis, showing outstanding catalytic performance in a variety of reactions^1,2^, including electrochemical reactions, water-gas shift reactions, and hydrogenation reactions. The SACs have shown a wide range of applications in synthetic organic chemistry^3^, solar hydrogen technologies^4^, and low-platinum fuel cells^5^.

The production of well-dispersed isolated atoms as heterogeneous catalysts remains challenging due to the high-surface energy of individual atoms. Various strategies have been developed for the fabrication of SACs. High-vacuum physical deposition technologies, such as atomic-layer deposition^6^, provide ideal model catalysts for fundamental studies of the metal–support interaction^7^. However, these high-vacuum methods are difficult for large-scale production due to the complex and costly equipment and low yield. Meanwhile, a variety of wet-chemical routes, including co-precipitation^8^, impregnation^9^, acid leaching^10^, and de-alloying^11^, are commonly used for catalyst synthesis. The adaptation of these methods in the production of SACs remains limited, for the complexity, poor versatility, long processing time, and high post-processing cost^12^. In addition, defect engineering^13^ and organic synthesis (e.g., metal–organic framework^14^) also suffer from poor versatility and tedious precursor modifications.

On the other hand, facile fabrication of heterogeneous SACs has been achieved by high-temperature pyrolysis of precursors on a wide variety of supports. For instance, a single atom Fe, Co, or Ni on carbon nanotube was obtained by multistep pyrolysis of the mixture of dicyandiamide with iron, cobalt, and nickel acetylacetonate, respectively^15^. Very recently, Yao et al. demonstrated the use of high-temperature shockwaves generated from pulsed electrical Joule heating of the H_2_PtCl_6_-loaded activated carbon nanofiber to synthesize and stabilize single Pt atoms^16^. In this research, proper controls of the heating period and temperature (~1500 K) are critical to avoid agglomeration and provide sufficient activation energy for the formation of a thermodynamically stable Pt–C bond. This synthetic method extends the previous high-temperature atom trapping and pyrolysis routes^17,18^, which improves processing efficiency and can be easily scaled up.

Lasers, as a non-contact manufacturing tool, benefit from various available wavelengths, high-energy-density, rapid scanning speed, high spatial resolution, and good flexibility in designing complex patterns and 3D architecture^19^. They have been used to fabricate various kinds of nanoparticles (NPs), including metal, alloy and metal oxide NPs, semiconductor quantum dots, and core-shell NPs^20,21^, offering a powerful and flexible alternative to the purely chemical approaches for the synthesis of NPs. Laser-induced synthesis of NPs mainly based on two different mechanisms. The first mechanism is based on laser ablation in liquid to produce colloidal NPs with particle size typically above 5 nm and broad size distribution^21^. The second mechanism is based on laser-assisted photochemical processes to generate metal (Au, Ag, and Pd) NPs down to sub-5 nm through photochemical reduction of metal salt precursors in aqueous solution, e.g., using ultrashort laser pulses to induce solvated electron from the liquid medium or excite electron–hole pairs in the semiconducting solute that are capable of reducing soluble metal ions to neutral atoms, which then coalesce into NPs^22,23^. However, the synthesis of atomically dispersed species via liquid-phase laser photo-deposition remains challenging, as the nucleation and crystal growth can hardly be prevented without effective confinement of the reactants.

In this paper, for the first time, we report a laser-induced solid-phase strategy for the synthesis of atomically dispersed Pt on graphene support by simultaneous pyrolysis of metal salt precursor and reduction/graphitization of electrochemical graphene oxide (EGO). The Pt-EGO samples for laser irradiation were prepared by freeze-drying the H_2_PtCl_6_ infused EGO hydrogel films to form “isolated dispersion” of H_2_PtCl_6_ precursor on the EGO substrate. In order to establish laser synthesis mechanisms, two types of lasers, namely, a picosecond (ps) pulsed ultraviolet (UV) laser with a wavelength of 355 nm and nanosecond (ns) pulsed infrared (IR) Nd:YAG laser with a wavelength of 1064 nm were used; and the temperature evolution during the laser irradiation was recorded. For the use of the ns IR laser, the localized temperature reached up to ca. 1350.2 K as measured by the thermal camera; finite element modeling suggested a peak temperature as high as 1692.6 K, suggesting thermal decomposition route via photothermal effect. After the laser irradiation, single Pt atoms decorated laser-reduced EGO (Pt-LrEGO) with a Pt loading of 0.41 wt.% were successfully synthesized. As electrocatalysts, the Pt-LrEGO presented a small overpotential of −42.3 mV at −10 mA cm^−2^ in hydrogen evolution reaction (HER) with a mass activity over ten times higher than that of the commercial Pt/C. This laser synthesis method is one-step, simple, ultrafast, and potentially scalable, which can be readily extended to other metals and supporting materials for mass production of functional SACs for various applications.

## Results and discussion

**Fig. 1:**
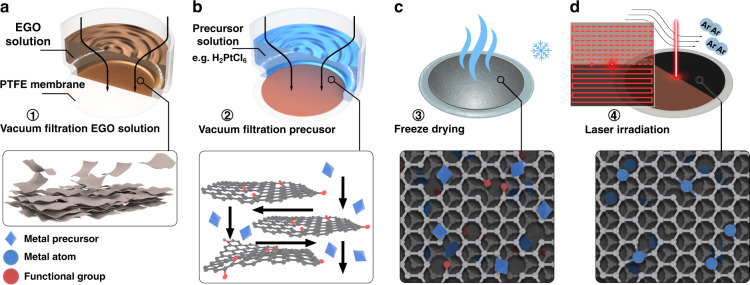
Schematic illustration of the formation of Pt-LrEGO. **a** Preparation of EGO film from 1.0 mg mL−1 EGO solution through vacuum filtration; **b** metal precursor filter through the EGO film and followed by (**c**) immediate freeze in liquid nitrogen and freeze-drying; **d** direct 1064 nm laser irradiation of the Pt-EGO to form Pt-LrEGO, inset shows the schematic of the parallel laser scanning path across the EGO film, the laser beam spot size was 1 mm in diameter and overlap ratio between the tracks was 50%

The laser synthesis is illustrated in both Fig. 1 and Supplementary Fig. S1. First, the EGO film was prepared by vacuum filtration of the EGO aqueous dispersion through a PTFE membrane filter, then 1 mL of the chloroplatinic acid (H_2_PtCl_6_) solution with desired concentration (1, 2, 3, 5, or 10 mM) was filtered through the EGO film to obtain the Pt-EGO hydrogel film. The freshly filtered, wet Pt-EGO hydrogel film supported by the PTFE membrane filter was immediately frozen in liquid nitrogen, followed by freeze-drying in a vacuum to sublimate the ice. The purpose of this direct sublimation of solid ice to water vapor was to immobilize the [PtCl_6_]^2−^ anion complex on negatively charged EGO flakes to avoid localized precipitation of the metal precursor. The as-prepared Pt-EGO films were subject to laser irradiation under Ar atmosphere via rapid scanning. After the laser treatment, the laser-reduced EGO (LrEGO) film loaded with Pt single atoms were separated from the PTFE membrane filter by sonication in water, followed by washing and freeze-drying to obtain the final materials in a powder state. The as-formed graphene-supported Pt SACs are denoted as Pt*x*-LrEGO, where *x* represents the concentration of the H_2_PtCl_6_ precursor solution filtered through the EGO film, e.g., Pt5-LrEGO represents the concentration of H_2_PtCl_6_ precursor solution is 5 mM, and Pt1-LrEGO was prepared by treating the EGO film with 1 mM H_2_PtCl_6_. To distinguish the IR laser-treated LrEGO, the UV-fabricated reduced graphene is denoted as Pt*x*-LrEGO_UV_.

### Characterization of Pt single atoms

**Fig. 2:**
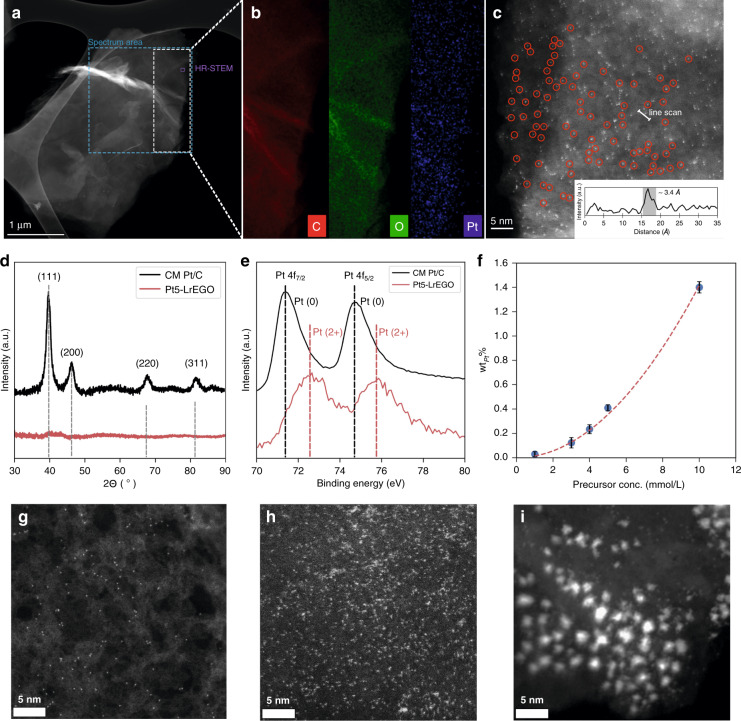
Structural characterizations of the Pt-LrEGO samples. **a** HAADF-STEM image of Pt-LrEGO after 1064-nm laser irradiation at a laser fluence of 7.66 mJ cm^-2^ and **b** EDX mapping of C, O, and Pt of the selected area. **c** Representative HAADF-STEM image showing isolated Pt atoms deposited on LrEGO support (inset shows the representative HAADF intensity profile). **d** XRD pattern and **e** XPS spectra of the commercial Pt and Pt5-LrEGO fabricated by 1064 nm laser at 7.66 mJ cm^−2^. **f** ICP-OES result of Pt loading within Pt-LrGO with various precursor concentrations, and representative STEM images of **g** Pt1-LrGO, **h** Pt5-LrGO, and **i** Pt10-LrGO processed at 7.66 mJ cm^−2^

The samples were characterized with the aberration-corrected high-angle annular dark-field scanning transmission electron microscope (HAADF-STEM). Figure 2a shows the HAADF-STEM images of the Pt5-LrEGO sample prepared by the ns IR Nd:YAG laser, revealing an intact 2D flake without the presence of Pt nanoparticles or clusters. Nevertheless, Energy-dispersive X-ray (EDX) elemental maps (Fig. 2b) indicate the presence of homogeneously dispersed Pt element over the entire LrEGO support. The HAADF-STEM image at high magnification (Fig. 2c) clearly shows the Pt element on the LrEGO support exists as extremely small bright dots with a diameter of ~0.34 nm, corresponding to the diameter of an individual Pt atom. The HAADF-STEM characterization clearly suggests the presence of single Pt atoms on the EGO support.

In addition to the HAADF-STEM characterization, X-ray diffraction (XRD) and X-ray photoelectron spectroscopy (XPS) further confirm that the Pt species exist in the Pt-LrEGO as uniformly dispersed single atoms. In detail, the XRD pattern of Pt5-LrEGO in Fig. 2d indicates the absence of crystallized Pt nanoparticles, as the characteristic peaks of Pt (111), (200), (220), and (311) (ICDD No. 00-004-0802) that clearly appear in the XRD pattern of commercially available (CM) Pt/C are missing in the XRD pattern recorded from the Pt5-LrEGO sample. XPS Pt 4f high-resolution spectra provide further evidence for the single atomic dispersion of Pt in the Pt5-LrEGO sample. As shown in Fig. 2e, the Pt 4f high-resolution spectra of the CM Pt/C show a set of doublet peaks located at 71.4 and 74.7 eV, corresponding to the doublet peaks of Pt (0) 4f_7/2_ and 4f_5/2_, respectively^24^. For the laser-treated Pt5-LrEGO, the doublet peaks shifted to the higher binding energy of 72.6 and 75.8 eV, corresponding to Pt (2+)^24^. The lack of detectable Pt (0) signal implies that the Pt nanoparticle/clusters were unlikely to exist or with a negligible content in the sample, conforming to the absence of crystallized Pt signals in the XRD patterns. The Pt (2+) originates from the interaction between the single Pt atoms and substrates^24^. The Pt loading for the Pt5-LrEGO is 0.41 ± 0.01 wt_._% as determined by inductively coupled plasma–optical emission spectrometry (ICP-OES).

### Effects of precursor concentration and precursor type

Variation in the concentration of H_2_PtCl_6_ solution filtered through the EGO hydrogel film showed a strong influence on the size of the as-formed Pt species. As shown in Fig. 2f and g, the laser-treated Pt1-LrEGO using the 1 mM of H_2_PtCl_6_ solution shows atomically dispersive Pt species in a similar size of ~0.34 nm but with a low Pt loading of ~0.03 wt.%. With the increase of the H_2_PtCl_6_ concentration to 5 mM, the Pt species remains atomically dispersed on the LrEGO flake (Fig. 2h) while the Pt loading increases to 0.41 wt.% without noticeable aggregation. Further increasing the concentration of H_2_PtCl_6_ to 10 mM led to clear aggregation of the Pt single atoms (Fig. 2i). The weight percentage of Pt species increased from 0.03 to 1.40 wt.% with H_2_PtCl_6_ concentration from 1 to 10 mM (Fig. 2f).

**Fig. 3:**
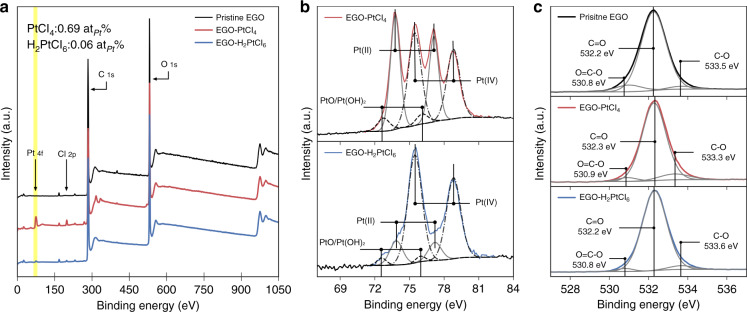
XPS characterization of the pristine EGO, and PtCl_4_ (5 mM) and H_2_PtCl_6_ (5 mM) filtered EGO films. **a** XPS survey, **b** high-resolution XPS Pt 4f spectra, and **c** O 1 s spectra

In addition to the concentration of precursor solution, the type of Pt precursor compounds, namely, H_2_PtCl_6_ or PtCl_4_, shows a significant impact on the as-formed Pt species on the LrEGO support. Unlike the H_2_PtCl_6_ solution that leads to atomically dispersed Pt on the LrEGO flakes, the STEM images (Supplementary Fig. S2) of the Pt-LrEGO synthesized from the PtCl_4_ precursor via the same laser parameters show prevailing the formation of Pt nanoparticles for all the concentrations used (from 1 to 10 mM). We performed XPS analysis for the precursor adsorbed EGO to understand the interfacial interaction between PtCl_4_, H_2_PtCl_6_, and the EGO support. Figure 3a presents the XPS survey of the pristine EGO, and EGO infused with PtCl_4_ (5 mM) and H_2_PtCl_6_ (5 mM) precursors. Except the C 1 s and O 1 s peaks that exist in all samples, the Pt 4f and Cl 2p peaks were found in both of the precursor infused EGO films. Through comparison of the integrated intensity of Pt, Cl, C, and O, the percentage of Pt species in the spectral are ~0.69 at_Pt_% and ~0.06 at_Pt_% for EGO-PtCl_4_ and EGO-H_2_PtCl_6_, respectively. The content of adsorbed Pt species on the EGO-PtCl_4_ is one-order magnitude higher than that of the EGO-H_2_PtCl_6_, clearly indicating the difference in interfacial interaction and adsorption tendency. Besides, in exploring the remaining Cl element within the samples, we found that the Pt:Cl ratio for EGO-H_2_PtCl_6_ is 1:5, which is close to that of the original compound, while the Pt:Cl ratio for EGO-PtCl_4_ is ~1:1.5. Figure 3b shows the Pt 4f spectra of the EGO-PtCl_4_ and EGO-H_2_PtCl_6_, each Pt 4f peak can be de-convoluted into two pairs of doublets and were assigned to Pt (IV) (PtCl_4_/H_2_PtCl_6_), Pt (II) (PtCl_2_) and PtO/Pt(OH)_2_ (binding energy summarized in Supplementary Table S1)^25,26^. Apart from the Pt (IV) peaks from the precursors, the existence of PtO/Pt(OH)_2_ peaks attributes to the hydrolysis of the metal salts by humid air^25^. The dominating Pt (II) components in the Pt 4f spectrum of EGO-PtCl_4_ are possibly caused by the reduction of Pt^4+^. Owing to the electrophilic nature of Pt (IV), the Pt (IV) can oxidize hydrocarbon and itself turns to PtCl_2_ and HCl^27^. In our study, the oxidation of EGO caused by PtCl_4_ is more pronounced than H_2_PtCl_6_, this explains the disproportion of Pt:Cl in the samples. By examining the O1s spectra (Fig. 3c), the integrated area of C–O peak of EGO-PtCl_4_ among the de-convoluted O1s signal is 11.6%, which is two times larger than 5.4% of EGO-H_2_PtCl_6_. Moreover, the binding energies of the C–O group are at 533.5, 533.3, and 533.6 eV for the pristine EGO, EGO-PtCl_4_, and EGO-H_2_PtCl_6_, respectively; the relatively smaller binding energy of the C–O group in EGO-PtCl_4_ provides evidence for the adsorption of Pt species on the epoxy and/or hydroxyl groups of EGO^26^.

The excellent hydrophilicity of EGO in water is largely due to the negatively charged oxygen-containing groups on the surface, where hydrated cation tends to interact with the oxidized groups through coupling between the oxygen atoms and the empty orbitals of metal cations^28,29^. The previous research^30^ on quantification of Pt uptake by oxidized carbon substrates indicates the amount of PtCl_6_^2−^ taken up was negligible compared to Pt(NH_3_)_2_^+^. Similarly, the positively charged Pt^4+^ from PtCl_4_ solution is most likely adsorbed on EGO via electrostatic interaction and forms EGO-Pt (II/IV) complex before freeze-drying, which might adversely affect the dispersion of Pt^4+^ on the EGO substrate, and thus unlikely to form Pt single atoms.

Therefore, the successful formation of single Pt atoms anchored on the LrEGO support in this case strongly relied on controlling the ion distribution on the supporting materials and the precursor concentration and types. Reducing the precipitation of metal precursors and physical increasing the distance of ions/single atoms thus suppress aggregation and formation of clusters/nanoparticles.

### Characterization of laser-reduced EGO

**Fig. 4:**
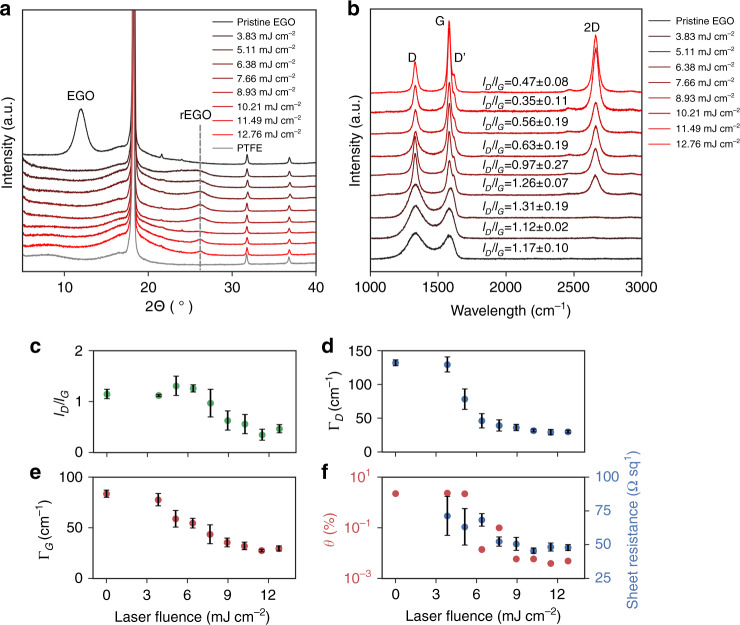
Characterizations of the Pt-LrEGO prepared with different laser fluences. **a** XRD patterns (the diffraction peaks centered at 2*θ* = 18.1°, 31.7°, and 36.8° in the XRD pattern originated from the PTFE membrane filter) and **b** representative Raman spectra of the pristine EGO and LrEGO after 1064 nm laser irradiation at various laser fluences. The evolution of **c** *I*_D_*/I*_G_ ratio, **d**, **e** FWHM values of D and G bands, and **f** defect density (*θ*) and sheet resistance of EGO and LrEGO samples

In addition to the formation of single Pt atoms, the quality of the supporting LrEGO also plays an important role in the catalytic performance by building up an electrical conduction network. Supplementary Fig. S3 shows FEG-SEM images of the Pt5-LrEGO prepared using the ns IR laser. A significant expansion of the EGO film was observed in the laser-irradiated area with the film thickness increased ~27 times from 1.3 to 35 μm, reflecting a drastic formation and release of gaseous products during the laser irradiation. The XRD patterns of the Pt5-LrEGO irradiated by the ns IR laser (Fig. 4a) show the disappearance of the characteristic diffraction peak of EGO at a 2*θ* = 11.9° for all the laser fluences (0−12.76 mJ cm^−2^, note: zero fluence represents pristine EGO) and the peaks centered at 2*θ* = 26.2°, representing (002) planes of restacked graphene, raised gradually with increasing laser fluence, indicating the occurrence of the reduction of EGO. Furthermore, XPS characterization suggests the oxygen contents of EGO dropped from 20.04 to 5.08 at.%, after laser irradiation. The XPS high-resolution C1s spectra collected from the pristine EGO film and the Pt5-LrEGO films after the IR laser irradiation at 7.66 mJ cm^−2^ reveal a significant decrease in oxygen functional groups. The deconvolution of the C1s spectrum of the pristine EGO shows four characteristic components, which are assigned to C1s (284.5 eV), C−O (286.4 eV), C=O (287.9 eV), and −COO− (289.2 eV), respectively (details in Supplementary Fig. S4 and Supplementary Table S2)^31^. In comparison, the C1s spectrum of the LrEGO film shows a significant decrease in the proportions of the oxygen components, and the π to π* peak at (290.8 eV) appears, showing an effective removal of oxygen groups and restoration of the graphitic structure.

Raman spectroscopy with an excitation wavelength of 633 nm was used to characterize the graphitization degree of Pt5-LrEGO, the acquired spectra were fitted with Lorentzian function after baseline subtraction for further analysis. Figure 4b shows the typical Raman spectra of the pristine EGO and LrEGO samples: the D band located at ~1332 cm^−1^ represents the edge planes and disordered structures; the G band at ~1581 cm^−1^ is induced by the ordered sp^2^ bonded carbon; the D‘ peak centered at ~1619 cm^−1^ is originated from an intravalley double resonance process; the peak at ~2660 cm^−1^ is assigned to the 2D band which is the second-order of the D peak^32,33^. To monitor the evolution of defect density in the Pt5-LrEGO sample after laser irradiation, the *I*_D_/*I*_G_ ratio as a function of irradiation fluence was plotted in Fig. 4c. The value of *I*_D_*/I*_G_ ratio is closely related to the density of defect/functionality within the graphene basal plane^33,34^. For the use of the ns IR laser as shown in Fig. 4c, the D and G bands intensity ratio increased initially from 1.17 ± 0.10 to 1.31 ± 0.19, followed by a drastic drop to 0.35 ± 0.11 with the increasing laser fluence. This evolution of *I*_D_/*I*_G_ ratio fits Ferrari and Robertson’s classification of the disorder transition from amorphous carbons to graphite^35^, indicating the decrease of structure disorder with the increase of laser fluence. The reduction and restoration of graphene structure in the Pt5-LrEGO were also supported by the observation of the narrowing of the full width at half-maximum (FWHM: Γ) of D and G bands with the increase of laser fluence (Fig. 4d, e). The model proposed by Cançado et al.^33^ was then applied to quantify the defect distance (*L*_D_) and defect density (*θ*) of the LrEGO films from the Raman *I*_D_/*I*_G_ ratios (Supplementary Table S3). Figure 4f shows the defect density retained ~2.25% at the laser fluence of 6.38 mJ cm^−2^, and then dropped precipitously and down to ~0.05% at a laser fluence of 7.66 mJ cm^−2^. This observation provides clear evidence for the efficient removal of oxygen groups along with the restoration of the graphene structure under the ns IR laser irradiation. The probe of the sheet resistance change after laser irradiation provides further evidence for the restoration of the electrically conductive sp^2^ structure. The sheet resistance of the Pt5-LrEGO film (Fig. 4f) decreases with the laser fluence from 3.83 to 6.38 mJ cm^−2^ and remains nearly constant at higher fluence up to 12.76 mJ cm^−2^. A minimum sheet resistance value of 45.6 Ω sq^−1^ was achieved at the laser fluence of 10.21 mJ cm^−2^. Note the pristine EGO is an insulator and the resistance value is beyond the instrument detection limit.

The Pt5-LrEGO_UV_ films treated by UV laser with a relatively narrower fluence range and denser pulse overlap conditions compared with the IR laser also show a similar trend in *I*_D_*/I*_G_ ratio, Г_D_ and Г_G_ values with the increase of laser fluence (Supplementary Fig. S5). The ordered crystalline structure occurred in a fluence range from 1.8 to 2.0 mJ cm^−2^. However, at a laser fluence below 1.8 mJ cm^−2^, the relatively high *I*_D_*/I*_G_ ratio and Г_D_ and Г_G_ values, together with the broadened standard deviation (Supplementary Table S4, statistical Raman analysis was based on 10 spectra recorded at random points of each sample), indicate the reduction degree of EGO is low and uneven. The poor reduction degree is also reflected by the sheet resistance of Pt5-LrEGO_UV_. Supplementary Fig. S5e shows that the Pt5-LrEGO_UV_ has decreased sheet resistances at increasing laser fluence, but with much less extent than that seen in the IR-irradiated LrEGO. The unevenness in reduction degree was further verified by XRD (Supplementary Fig. S6). The Pt5-LrEGO_UV_ films treated by the ps UV laser show a clear peak at 2*θ* = 11.9°, and the peak intensity gradually decreased and completely disappeared when the laser fluence reached 2.0 mJ cm^−2^, while the graphite (002) peaks increased progressively at the laser fluence exceeding 1.45 mJ cm^−2^. When the laser fluence reached 2.09 mJ cm^−2^, the EGO film was ablated completely from the PTFE membrane.

Taken together, the Raman, XRD, and sheet resistance results strongly suggest the photoreduction of EGO through IR and UV radiation occurs in different photoinitiated manners. From a practical perspective, efficient and even reduction of EGO film was found in IR irradiation, which is evidenced by the high crystallinity and low sheet resistance.

### SACs laser synthesis mechanisms

The laser synthesis introduced in this work was based on photoreduction of the metal precursor and EGO simultaneously. Unlike other chemical and thermal reduction methods, laser reduction is characterized by energy absorption by the GO films dependent on the radiation wavelength and pulse width. From the wavelength perspective, the photoreduction of GO generally can be achieved photochemically with radiation wavelength in the UV region^36^ and photothermally using radiation from visible to IR^37^. From the temporal perspective, the laser–matter interaction occurs in a clear time sequence^38^: excitation of an electron by incident photon occurs in the first ~100 fs, following by electron–phonon coupling from 100 fs to 10 ps, finally from 100 ps or longer heat dissipate to the surrounding matrix. Therefore, thermal analysis is invalid for fs laser irradiation. In our current study, the pulse width of 5 ns is sufficient for heat conducting to the bulk materials.

**Fig. 5:**
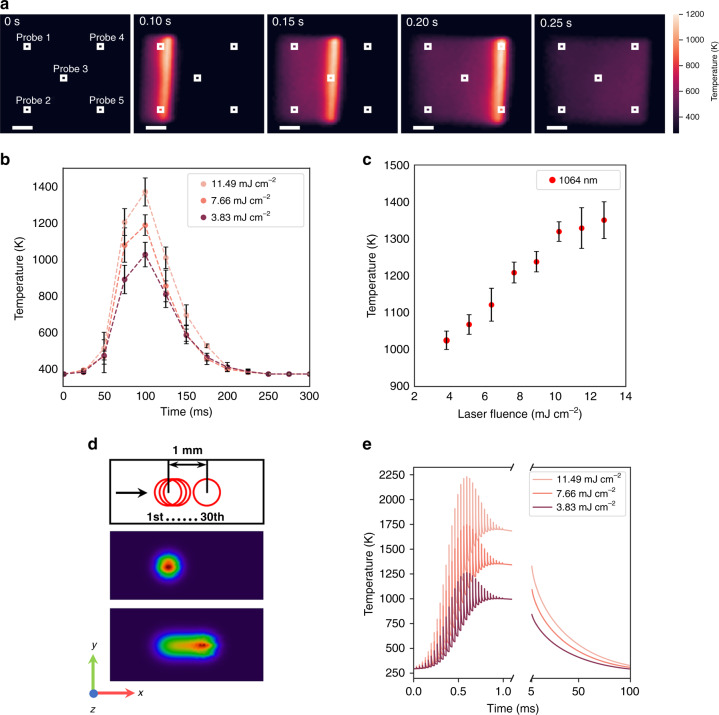
Thermal profiles of the EGO films under irradiation with different laser fluences. **a** The infrared images of EGO under 1064 nm laser irradiation at an average power of 6 W and at a scanning speed of 1000 mm s^−1^ (the scale is 2.5 mm); **b** typical temporal thermal profiles at an incident laser fluence of 3.83, 7.66, and 11.49 mJ cm^−2^, respectively. **c** The change of the measured peak temperatures with the variation of laser fluences under at a scanning speed of 1000 mm s^−1^. **d** Schematic of 30 successive laser pulse train irradiated on the EGO surface in COMSOL modeling, and the temperature fields on the top surface of EGO film after the first and the thirtieth pulse irradiation. **e** COMSOL modeling of the EGO film processed by 1064 nm laser at various laser fluences

When the ns IR laser with a photon energy of 1.17 eV irradiated on the EGO surface, the absorbed laser energy was rapidly converted into local heat via photothermal effect and raise the local temperature, depending on the laser-processing conditions i.e., laser fluence and number of pulses per point (NOPs) determined by the beam size, scanning speed, and repetition rate. To evaluate the temperature profile of EGO film under laser irradiation, we conducted thermal camera measurement of EGO to record the temperature images of EGO scanned by 1064 nm laser at various fluences. Figure 5a shows the thermal images of the EGO surface captured at different stages of the laser scanning process at the laser fluence of 7.66 mJ cm^−2^. To compare the temperature evolution of the EGO surface at different laser fluences, we monitored the temperature evolution at five probe points (as marked in Fig. 5a) for each laser fluence. By averaging the time trace temperatures of these five probe points (see details in Supplementary Fig. S7), we obtained the representative temperature-time profiles for each laser fluence. Figure 5b shows the temperature evolution of the EGO surface at the laser fluences of 3.83, 7.66, and 11.49 mJ cm^−2^. Figure 5c compares the peak temperatures at various laser fluences under the same NOP (i.e., 30). It is obvious that the increase of the laser fluences results in the increase of temperatures up to 1350.2 ± 50 K at the laser fluence of 11.49 mJ cm^−2^. In addition, due to the high repetition rate (30 kHz), the heat spike induced by each pulse was not able to cool down to the initial temperature between pulses. Therefore, the heat accumulation results in surface temperatures being over 493.2 K for a period up to ~150 ms. However, owing to the fast temperature change and limited frame rate of the infrared camera, the observed time trace temperature profiles was a result of heat accumulation rather than the actual heating pattern.

In order to reveal the thermal evolution, COMSOL Multiphysics software was used to simulate the EGO film temperature dynamics undergoing 1064 nm laser irradiation at varying laser fluences. Owing to the extremely small duty cycle of 1/6660 (5 ns, 30 kHz), the calculation of the entire process requires tremendous computing time. Thus, the simulation was conducted by depositing 30 successive pulses scanned across the surface probe at 1000 mm s^−1^ with an interval of ~33.3 µs (Fig. 5d). Each individual pulse possesses a Gaussian energy distribution and has a laser ON time of 5 ns. The simulated temporal thermal profile shown in Fig. 5e shows a short heating time of ~1 ms to reach up to 1692.2 K, and a fast thermal relaxation to 298.15 K within ~108 ms. At a given laser fluence, the rapid heating and cooling during and after the laser ON period were clearly shown, and the peak temperature gradually rises with increasing accumulated heat and spatial energy density. When the laser beam bypassed the probe, the accumulated heat reached the highest value of 998.9, 1348.2, and 1692.6 K which corresponds to the laser fluences of 3.83, 7.66, and 11.49 mJ cm^−2^, respectively.

As described in Schweizer and Kerr’s work^39^, the thermal breakdown of H_2_PtCl_6_ to form platinum metal takes place through several steps as follows: H_2_PtCl_6_ → PtCl_4_ + 2HCl at 423.2–453.2 K, PtCl_4_ → PtCl_2_ + Cl_2_ at 574.2–594.2 K, PtCl_2_ → Pt + Cl_2_ at 648.2–783.2 K. Therefore, the temperature of 1025 K achieved during the 1064 nm laser irradiation at the lowest laser fluence (3.83 mJ cm^−2^) was sufficient to induce in situ thermal decomposition of the adsorbed H_2_PtCl_6_ molecules in the EGO film to form Pt single atoms. The rapid heating and cooling during the laser processing inhibit the diffusion and aggregation of the Pt atoms, and the short thermal pulse at temperatures up to 1692.2 K is likely to promote the formation of stable bonding between the Pt single atoms and the graphene support. Interestingly, at the H_2_PtCl_6_ concentration of 5 mM, the as-formed Pt species remain as atomically dispersed single atoms with the increase of laser fluence from 3.83 to 11.49 mJ cm^−2^ (Supplementary Fig. S8), indicating that the rise in temperature had not promoted the diffusion of the single atoms, which suggested stable metal–substrate bonding and high stability of the atomic dispersion.

This can be further supported by the consideration of two types of overlapping involved in the beam scanning. One is that as described in Fig. 1, the laser beam spot size was 1 mm in diameter and overlap ratio between the tracks was 50%. This means that 50% of the laser track with already formed Pt single atoms was re-treated and a portion adjacent to the successive track was re-heated. However, no Pt aggregation/clusters were evident. The other type of overlapping is always present in pulsed-laser scanning. For instance, with the beam size of 1 mm, the scanning speed of 1000 mm/s and repetition rate of 30 kHz, the number of pulses per point (NOPs) is 30, which means that the Pt single atoms were exposed to the successive pulses, but no Pt aggregation was observed. This further indicated the stability of the Pt single atoms once synthesized and anchored to the supporting material was formed. On the other hand, thermal reduction of EGO involves the removal of functional groups and restoration of carbon structures. Although the reduction of carboxyl groups with low stability starts at 100–150 °C^40^, total elimination of all the functional groups including highly stable carbonyl groups needs a high temperature (>1000 °C)^41^, and carbon structural evolution is temperature-dependent. During the laser irradiation, the temperature range achieved was sufficient to induce the photothermal reduction of the EGO, and the re-heating caused by overlapping should be beneficial to the reduction degree.

In order to investigate the effect of the laser irritation time, the scanning speed was set to be 800 mm/s with the same laser fluence to 7.66 mJ cm^−2^ to avoid damage of the EGO layer. In this case, the Pt particles/clusters were found in Supplementary Fig. S9, implying that the duration, which also enhances the degree of overlapping, played an important role on atomic diffusion/aggregation of Pt single atoms.

When the ps UV laser was applied, photochemical removal of functional groups was expected^42^. The photon energy of the 355 nm light is 3.49 eV can excite single-photon-mediated valence-to-conduction band transition of EGO (with a direct bandgap range from 3.25 to 3.95 eV)^36,43^. While for the 1064 nm beam at a photon energy of ~1.17 eV, multiphoton process is required to excite EGO. However, the peak temperature raises of the EGO surface monitored by the thermal camera (Supplementary Fig. S10) reached 803.7 K at the laser fluence of 2.0 mJ cm^−2^ and the number of pulses of ~404, suggesting that both photochemical and photothermal heating took place simultaneously. Since the UV laser (355 nm, ~4.86 eV) photon energy is far below the resonant excitation of C–O or C=O bond^22^, direct excitation may occur through valence-to-conduction band transitions, which may lead to ejection of plasma in the material^43^ and/or photoreduction of EGO film by photon excited electrons of residual water molecules^22^ or EGO itself^36^. As the direct ionization occurs ahead of thermal accumulation^43,44^, the photoreduction of the EGO could occur via combined mechanisms with the following sub-processes: (i) removal of oxygen-containing functional groups, (ii) conversion of reduced carbon sp^3^ into sp^2^ structure, and (iii) restoration of sp^2^ network. The removal of functional groups and the conversion of sp^2^ structure can be achieved by both photochemical and photothermal routes, but the restoration of sp^2^ network might be dominated by thermal effect^45,46^. As demonstrated in Supplementary Figs. S5e and 4f, the defect density of the LrEGO by the ps UV laser was higher than that by the ns IR laser, indicating the higher temperature in the ns IR laser irradiation might benefit the restoration of sp^2^ carbon network.

In addition, the EGO films contain multiple layers of EGO flakes, one of the reasons of the nonuniform reduction of EGO under UV laser irradiation might be from anisotropy of thermal conductivity in two-dimensional materials; and GO has over 100-fold anisotropy of heat flow between the in-plane and out-of-plane directions^47^. For an ultrashort pulsed laser in the ps or fs region, thermal diffusion volume is smaller than that of an ns laser, which results in a superficial reduction of EGO film with poor restoration of sp^2^ carbon^42^. Furthermore, laser beam conversion into thermal energy depends on not only the wavelength, pulse width, but also the stacked layers^48^. The penetration depth of the UV laser is smaller than that of the IR laser, and the ns pulse width also enhances heat conduction deeper than the ps pulse width. Thus, a thoroughly thermal reduction of EGO film can be expected when using ns IR irradiation. This was reflected by the XRD patterns (Supplementary Fig. S6) that the signals from the EGO were still present for the ps UV-treated Pt5-LrEGO, particularly for the low end of the laser fluences. This was also supported by the observation of the large error bars from ten measurements for various Raman parameters (Supplementary Fig. S5), suggesting that less uniform and smaller penetration of heating by the ps UV laser. Therefore, despite the effective removal of oxygen via photochemical dominating mechanism by the UV laser, reduction and structure restoration of the beneath layers are most likely achieved by thermal conduction with limited heat transport. Increasing the laser fluence of the ps UV laser caused ablation of EGO which accompanies endothermic phase changes and further impedes heat conduction. It could be concluded that the ns IR laser irradiation promotes an effective and uniform reduction of EGO throughout the EGO film in the thickness of 1.3 µm via a photothermal route.

In comparison to other chemical and physical methods in the fabrication of SACs, the laser photoreduction method is a one-step process, ultrafast laser manufacturing technique for the fabrication of Pt single atoms on the LrEGO support. Since the processing temperature by the ns IR laser can readily reach over 1350.2 K, which is sufficient to thermally decompose most of the metal precursors.

### Electrocatalytic performance

**Fig. 6:**
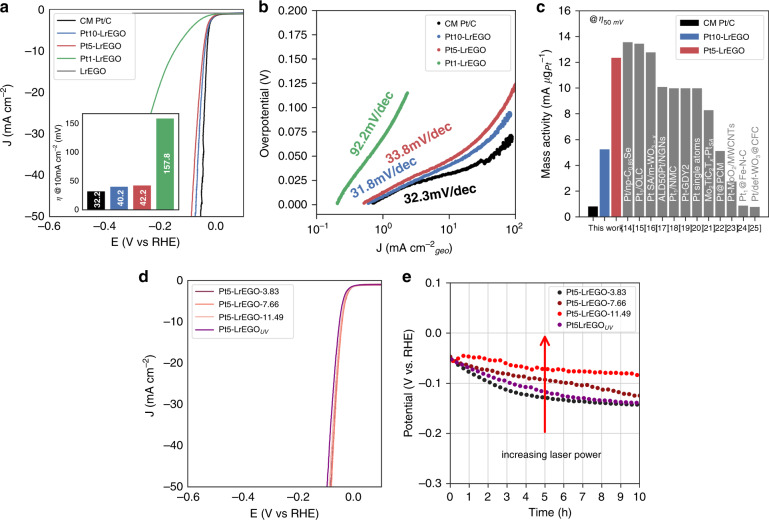
Hydrogen evolution performance of the Pt-LrEGO samples. **a** LSVs (10 mV s^−1^; after ohmic-drop correction) of the commercial Pt/C (20 wt_Pt_%) and the Pt-LrEGO catalysts prepared by laser irradiation (1064 nm; 7.66 mJ cm^−2^) of the EGO films infused with Pt precursor solutions at different concentrations; the LSVs were recorded in N_2_-saturated 0.5 M H_2_SO_4_ at a rotation speed of 1600 rpm; the inset shows the overpotential of samples at a current density of 10 mA cm^−2^. **b** Tafel slopes of the commercial Pt/C and Pt10-LrEGO, Pt5-LrEGO, and Pt1-LrEGO. **c** Mass activities of the commercial Pt/C, Pt-LrEGO, and the literature reported Pt-based SACs at an overpotential 50 mV. The bibliographic information of the reference numbers can be found in Supplementary Table S6 in Supporting information. **d** LSV of Pt5-LrEGO samples obtained by 1064 nm laser irradiation at various laser fluences. **e** Chronopotentiometry of Pt-LrEGO samples treated at various laser fluences at a current density of 10 mA cm^−2^

The electrocatalytic HER activities of the Pt-LrEGO samples treated by the ns IR laser was evaluated using a typical three-electrode system with a rotating disk electrode (RDE). The measurement was conducted in a N_2_-saturated 0.5 M H_2_SO_4_ electrolyte at a rotating speed of 1600 rpm. To reveal the intrinsic catalytic performance, the ohmic-drop correction was conducted in order to minimize the solution resistance. The linear sweep voltammogram (LSV) curves of the purchased commercial Pt/C (20 wt.%; Johnson Matthew, Highspec3000), LrEGO and Pt-LrEGO samples with varying Pt loading are shown in Fig. 6a. Note that the Pt1-LrEGO and Pt5-LrEGO have Pt species in the form of single atoms, while the Pt species in the Pt10-LrEGO present as clusters. The results show that the incorporation of Pt into LrEGO in a form of either single atoms or clusters enhances its HER activity, as the pure LrEGO exhibits negligible current change under the applied voltage of −0.3 V vs RHE. Using the thermodynamic HER potential (H^+^/H_2_ = 0 V vs RHE) as a reference, the Pt10-LrEGO and Pt5-LrEGO exhibited small overpotentials of 40.2 and 42.2 mV to drive a cathodic current density of10 mA cm^−2^, respectively, which are comparable to that of the commercial Pt/C catalyst (32.2 mV). With the further decrease of Pt loading to ~0.03%, the Pt1-LrEGO shows a large HER overpotential of 157.8 mV at 10 mA cm^−2^, indicating the lack of active sites on the LrEGO support.

Electrochemical impedance spectroscopy (EIS) was performed at an overpotential of 30 mV to evaluate the electrode kinetics for HER (Supplementary Fig. S11)^6,49^. The Nyquist plot indicates the Pt5-LrEGO possesses a low charge-transfer resistance (*R*_ct_) of 9.88 Ω implying a fast Faradaic process between the catalysts and electrolyte has been achieved. The HER catalysis kinetics of all samples were assessed by Tafel plot, as shown in Fig. 6b. The resultant Tafel slope of Pt10-LrEGO (31.8 mV dec^−1^) and Pt5-LrEGO (33.8 mV dec^−1^) are comparable to the CM Pt/C (32.3 mV dec^−1^), signifying superior HER kinetics in spite of the extremely low Pt loading (1.4 wt_Pt_.% and 0.41 wt_Pt_.% vs 20 wt_Pt_.%). As expected, the Pt1-LrEGO shows a large Tafel slope of 92.2 mV dec^−1^ due to the lack of active sites.

The mass activities of the catalysts were evaluated by normalizing the measured current at a given overpotential to the Pt mass loading, and an overpotential of 50 mV was chosen to compare with the literature reported values. As shown in Fig. 2f, because of the significantly lower Pt loading in the Pt5-LrEGO (0.41 wt_Pt_.%) compared to the CM Pt/C (20 wt_Pt_.%), the Pt5-LrEGO shows an order of magnitude higher mass activity (12.36 mA µg_Pt_^−1^) than the CM Pt/C (0.83 mA µg_Pt_^−1^). This superior HER mass activity of Pt5-LrEGO outperforms the majority of literature values (Fig. 6c, a detailed comparison is given in Supplementary Table S6), demonstrating ultrahigh Pt utilization and great promise for practical applications. Compared to the Pt5-LrEGO, the Pt10-LrEGO shows a moderate enhancement in mass activity, which is likely because of a fraction of inert Pt atoms embedded inside the clusters.

We subsequently studied the influence of laser fluence on the HER performance of the Pt single atoms decorated LrEGO. The Pt5-LrEGO treated by the ns 1064 nm laser at 3.83, 7.66, and 11.49 mJ cm^−2^ are denoted as Pt5-LrEGO-3.83, Pt5-LrEGO-7.66, and Pt5-LrEGO-11.49, respectively, and sample fabricated via ps 355 nm laser-irradiated at 2.00 mJ cm^−2^ sample is named as Pt5-LrEGO_UV_. Figure 6d shows the LSVs of above-mentioned catalysts tested in 0.5 M H_2_SO_4_ electrolyte, the overpotential at 10 mA cm^−2^ of Pt5-LrEGO-3.83, Pt5-LrEGO-7.66, and Pt5-LrEGO-11.49 were 43.3, 42.2, and 43.1 mV, respectively, demonstrating the HER activity and kinetics remain unchanged with various laser outputs under this fabrication condition. The Pt5-LrEGO_UV_ sample shows a slightly increased overpotential of 52.6 mV at 10 mA cm^−2^, possibly owing to the unevenly reduced LrEGO, which leads to a relatively larger charge-transfer resistance (*R*_ct_ = 14.2 Ω at an overpotential of 30 mV, Supplementary Fig. S11). Chronopotentiometric test at 10 mA cm^−2^ has been performed to evaluate the HER durability of Pt5-LrEGO samples. As shown in Fig. 6e, the Pt5-LrEGO-11.49 maintains a stable HER performance with a slight increase of overpotential by 39 mV after the 10 h testing. Nevertheless, the HER activity of Pt5-LrEGO-7.66 and Pt5-LrEGO-3.83 degrade remarkably after 10 h with a final potential of −0.1 and −0.13 V vs RHE to drive 10 mA cm^-2^ current density, indicating the Pt5-LrEGO HER durability increased with increasing laser fluence. STEM images acquired from the Pt5-LrEGO samples after the durability test are shown in Supplementary Fig. S12. By comparing Supplementary Fig. S8c and Supplementary Fig. S12a, b, there is only a slight increase in Pt size due to aggregation for the Pt5-LrEGO-11.49 after the durability test. This result implies a better durability of Pt5-LrEGO-11.49 SAC in the HER. In contrast, the Pt atoms coalesced into clusters and larger particles in Pt5-LrEGO_UV_, Pt5-LrEGO-3.83, and Pt5-LrEGO-7.66 (Supplementary Fig. S12c–h). The growth of the Pt active species might be ascribed to the migration of Pt atoms during the reaction because of the relatively weak atom–substrate interaction.

In addition, the emergence of H_2_ bubbles involved in the HER process can potentially cause damage to the basal plane of graphene^50^. The conductive pathways of 3D-interconnected graphene substrates will be destroyed after long-term testing, thus leading to the degradation of electrocatalytic activity in HER. Therefore, graphene substrate with less structural defects and better mechanical property is expected to exhibit superior stability during the HER test. According to the Raman results mentioned above, the resultant LrEGO shows fewer structural defects at higher laser fluences. Therefore, LrEGO obtained at a higher fluence can bear the hydrogen evolution perturbation well. This is in good coincidence with the stability test mentioned above. In addition, high-temperature treatment (>1173 K) can facilitate the stability of single atoms through a strong atom–substrate interaction, which potentially inhibits the migration and detachment of anchored atoms during HER^16^.

## Conclusions

In summary, for the first time, we have developed a rapid, versatile, solid-phase laser manufacturing technique for synthesizing graphene-supported Pt SACs. In this approach, the sublimation of the precursor solution contains metal anionic complex (PtCl_6_^2−^) promotes the "isolated dispersion" of H_2_PtCl_6_ precursor on the EGO substrate and prevents the localized aggregation by minimizing the electrostatic interaction between EGO and the metal ions. The pronounced photothermal effect of the 1064-nm laser irradiation in combination with fast scanning enables a high processing temperature (up to 1692.2 K) with a short heating time (~1 ms) and a rapid quenching time of ~108 ms, which not only guarantee the through-thickness thermal reduction of both EGO and Pt ions but also prevents the migration of metal atoms. We also demonstrated that the Pt-LrEGO exhibits promising activity and high Pt utilization in HER with an overpotential of 42.3 mV at 10 mA/cm^2^ and mass activity of 12.36 mA/µg_Pt_ at 50 mV. This laser-enabled solid-phase method potentially opens up a robust and versatile avenue for the production of a variety of single atoms on different substrates. Furthermore, this fast laser fabrication technique holds a promising potential in continuous roll-to-roll processing and could be applied in high-throughput catalyst synthesis and screening.

## Experimental section

### Materials

Electrochemical graphene oxide (EGO) was prepared using the previously reported two-step electrochemical intercalation and oxidation method^31^. Chloroplatinic acid hydrate (H_2_PtCl_6_·xH_2_O, 99.995%), platinum(IV) chloride (PtCl_4_, 99.99%), Nafion (5% in lower aliphatic alcohols and water) were purchased from Sigma-Aldrich and used as received. Platinum (1000 μg ml^−1^) ICP standard solution was purchased from SPEX CertiPrep. All chemicals were used as received without further purification, except dilution using deionized water (15 MΩ cm).

### Preparation of LrEGO composites film

To prepare the EGO film, 4 mL of 1 mg mL^−1^ EGO water dispersion was vacuum filtered through a PTFE filtration membrane (0.2-μm pore size, Millipore), the dimension of the EGO film was 3.2 cm in diameter and 1.5 ± 0.2 μm in thickness. In the case of Pt-EGO film, 1 mL metal precursor solution (H_2_PtCl_6/_PtCl_4_, 0.1–10 mmol L^−1^) was vacuum filtered through the EGO membrane. The EGO or Pt-EGO films were then immersed in liquid nitrogen and freeze-dried to reduce localized precipitation of metal salts. The dried GO and Pt-EGO films were subjected to direct laser beam patterning in constant Ar flow (Supplementary Fig. S1). Two laser sources were used to study the temperature-dependent SAC synthesis and the electrolysis stability: an infrared laser system of 1064 nm central wavelength (IPG Photonics, USA), 5 ns pulse width, 30 kHz repetition rate, and average power 3–10 W was focused into the EGO film with a spot size of ~1 mm. an ultraviolet laser system (355 nm, 10 ps pulse duration, EdgeWave GmbH, Germany) with pulse energy up to 54.4 μJ, a repetition rate of 404 kHz, a spot size of ~1 mm. A raster scanner (Fig. 1d) with a line spacing of 0.5 mm, which equals to the radius of the laser spot, was scanned across the Pt-EGO film in a parallel manner with a pattern dimension of 10 mm × 10 mm. The Galvo head scanning speed of both lasers was set to be 1000 mm/s.

The Pt-LrEGO were peeled off from the filtration membrane, the collected film samples were then sonicated in water, repeatedly washed (in deionized water, three times), and finally freeze-dried to obtain the catalyst powder for further electrochemical experiments.

### Materials characterization

The LrEGO films after laser irradiation were characterized using scanning electron microscopy (SEM) performed on a field emission Zeiss Ultra 55 SEM operating at 3 kV in lens mode. The high-resolution scanning transmission electron microscopy (HR-STEM) images and energy-dispersive X-ray spectroscopy (EDS) of Pt-LrEGO were captured by a C_s_-corrected FEI Titan G2 80–200 S/TEM ChemiSTEM operating at 200 kV equipped with a high-efficiency Super-X EDS detector system. To prepare TEM samples, the samples were dispersed and sonicated in ethanol as a dilution solution, then drop-casted onto the copper grid with a lacy carbon film. Raman spectroscopy was conducted using a Renishaw InVia Raman spectrometer, with a laser wavelength of 633 nm and a spot size of 2 µm; Raman spectra were analyzed using Wire 4.2 software, with Lorentzian function fitting after baseline subtraction. The peak intensity ratio of Raman D band to G band (*I*_D_*/I*_G_) for EGO and rEGO was obtained from the fitted peak intensities. The full width at half-maximum (FWHM; Г) of D and G bands was directly obtained from the curve fitting results. X-ray diffraction (XRD) were performed using a Proto AXRD benchtop powder X-ray diffractometer (XRD) with a Cu anode (*λ* = 1.5406 Å) operating at 30 kV. X-ray photoelectron spectroscopy (XPS) was performed with a SPECS NAP-XPS system; XPS spectra curve fittings were accomplished by CasaXPS software. The time-dependent temperature profile of the samples under laser irradiation was recorded using a FLIR T650sc infrared camera with a measurement range up to 2000 °C. The calibration of the infrared camera was conducted by the following steps^51^: an EGO film was adhered on a 3 M black electrical insulation tape and attached to a hotplate (Fisherbrand Isotemp, Fisher Scientific). The temperature was measured by a thermocouple (Model 88106-IEC, Omega) and controlled by the hotplate. The emissivity was calibrated through the infrared camera until the IR temperature is in accordance with the thermocouple measurement. The temporal temperature profile was recorded by averaging five individual points in each measurement. Four-point probe resistance measurements of the GO and LrEGO films were carried out using a four-point probe system (Janedel Engineering Ltd., Linslade, UK) equipped with a Keithley 2182 A nanovoltmeter and a Keithley 6220 current source (Keithley Instruments, Cleveland, OH, USA). To determine the mass of metal content within the Pt-LrEGO catalysts, an inductively coupled plasma–optical emission spectrometry (ICP-OES) was conducted by using a Analytikjena PlasmaQuant 9000 Elite system. All samples were weighed and digested in aqua regia for two days, the solution was then diluted and filtered with a 0.2-μm pore size Whatman syringe filter (Fisher Scientific, UK).

### Finite element (FE) simulation

The time-dependent FE simulation of the pulsed-laser patterning process on the EGO film was performed using COMSOL modeling to acquire a better understanding of the effects of laser fluence and NOPs on the temporal temperature elevation. An EGO film with 2 µm thick and dimensions of 1 cm by 1 cm was modeled as a candidate material, where its temperature-dependent material properties are acquired by taking typical values from literature (Supplementary Table S5). The pulsed laser with a Gaussian power density distribution is modeled as a heat flux on the surface of the EGO film. The heat transfer within the materials is governed by the COMSOL built-in general heat equation:

1 $$\rho C_p\frac{{\partial T}}{{\partial t}} + \rho C_pu \cdot \nabla T + \nabla \cdot \left( { - k\nabla T} \right) = Q$$

where the first, second, and third terms on the left-hand side are the time derivative of thermal energy per unit volume, convection energy, and conduction energy, respectively. *T* is the local temperature, *t* is the time span, *Q* is the converted energy from the laser beam to the material through photothermal conversion. *ρ*, *C*_*p*_, and *k* are mass density, specific heat capacitance, and thermal conductivity, respectively. The upper boundary of the film was set to be exposed to the boundary conditions of heat flux, natural convection, and irradiative convection. The lower boundary was subjected to an insulated boundary.

The pulsed-laser heat source with a Gaussian profile is shown in the following equation^38,48^:

2 $$Q = A \cdot P \cdot {\mathrm{Beam}}\left( {x,y,t} \right) \cdot {\mathrm{Pulsetrain}}\left( t \right)$$

where *A* is the absorptivity at a given incident radiation wavelength, *P* is the laser output power density, Beam (*x,y,t*) describes the Gaussian laser beam shape and its displacement, and Pulsetrain (*t*) describes the laser time-dependent pulse parameter. The Gaussian power density distribution of the laser beam was described as the following expression:

3 $${\mathrm{Beam}}\left( {x,y,t} \right) = \exp \left\{ { - \frac{{[x - \left( {x_0 - v_xt} \right)]^2}}{{2\phi ^2}} + \frac{{y^2}}{{2\phi ^2}}} \right\}$$

where x and y are the coordinates of the laser beam center, v_x_ is the beam travel velocity along the *x* direction, *t* is the time span, and *ϕ* is the standard deviation of the Gaussian laser beam. The thermal and optical properties of EGO used in the calculation are listed in Supplementary Table S5.

### Electrochemical experiments

All electrochemical experiments were performed in a conventional three-electrode system at a VersaSTAT4 Potentiostat (AMETEK, USA) and a 636 A electrode rotator system (AMETEK, USA) with an Ag/AgCl (saturated KCl solution) electrode as the reference electrode, a coiled platinum wire as the counter electrode, and a glassy carbon rotating disk electrode (RDE, Pine research) with a diameter of 5 mm (0.196 cm^2^) as the working electrode. All potential was converted to the RHE by using the following equation: *E*_RHE_ = *E*_Ag/AgCl_ + 0.197 + 0.059 pH. The RDE was polished with a microfiber polishing cloth with 0.05-μm alumina slurry until the mirror finish prior to all experiments. In all, 10 mg of catalyst was mixed with deionized water, isopropanol (99%, Aldrich), and Nafion (5%, Aldrich) with a volume ratio of 9:10:1, respectively, to a final concentration of 5 mg mL^−1^. After ultrasonication in an ice bath for 1 h, 10 μL of homogeneous ink was drop-casting on the RDE and dried under ambient, with a final loading of 50 μg (~0.255 mg cm^−2^). The Ohmic losses within the system were compensated by applying *IR*-correction. The uncompensated system resistance was determined by electrochemical impedance spectroscopy (EIS) at the open-circuit potential. The EIS was measured in a range of 100 kHz to 1 Hz, with a perturbation of 10 mV. The system resistance was then determined at the x-intercept of the Nyquist plot. The Nyquist plot during HER was based on EIS measurements at an overpotential of 30 mV in a frequency range of 10^–2^ to 10^6^ Hz with 10 mV sinusoidal perturbations in 0.5 M H_2_SO_4_. Linear sweep voltammetry with a scan rate of 10 mV s^−1^ was conducted in N_2_ saturated 0.5 M H_2_SO_4_ at a rotating speed of 1600 rpm. To obtain the current density, the current was normalized by the geometric electrode area, which is ~0.196 cm^2^. Chronopotentiometry measurements (at  10 mA cm^−2^) were conducted to evaluate the long-term HER stability.

## Supplementary information

Supplemental Material: Laser Solid-Phase Synthesis of Single-atom Catalysts
