# Supplementary material for: Laser solid-phase synthesis of single-atom catalysts
Source: Light Sci Appl. 2021 Aug 18;10:168. doi: 10.1038/s41377-021-00603-9 (PMC8373902; doi:10.1038/s41377-021-00603-9)
Supplement: Supplementary file 1 — Supplemental Material: Laser Solid-Phase Synthesis of Single-atom Catalysts [file 41377_2021_603_MOESM1_ESM.docx]

Supporting information

**Laser Solid-Phase Synthesis of Single-atom Catalysts**

*Yudong Peng^1^, Jianyun Cao^1,2^, Yang Sha^1^, Wenji Yang^1^, Lin Li^3^, Zhu Liu^1*^*

^1^Department of Materials, School of Natural Sciences, The University of Manchester, Oxford Road, Manchester, M13 9PL, UK

^2^National Graphene Institute, The University of Manchester, Oxford Road, Manchester, M13 9PL, UK.

^3^Laser Processing Research Centre, Department of Mechanical, Aerospace and Civil Engineering, The University of Manchester, Oxford Road, Manchester, M13 9PL, UK.

# Supporting figures

**
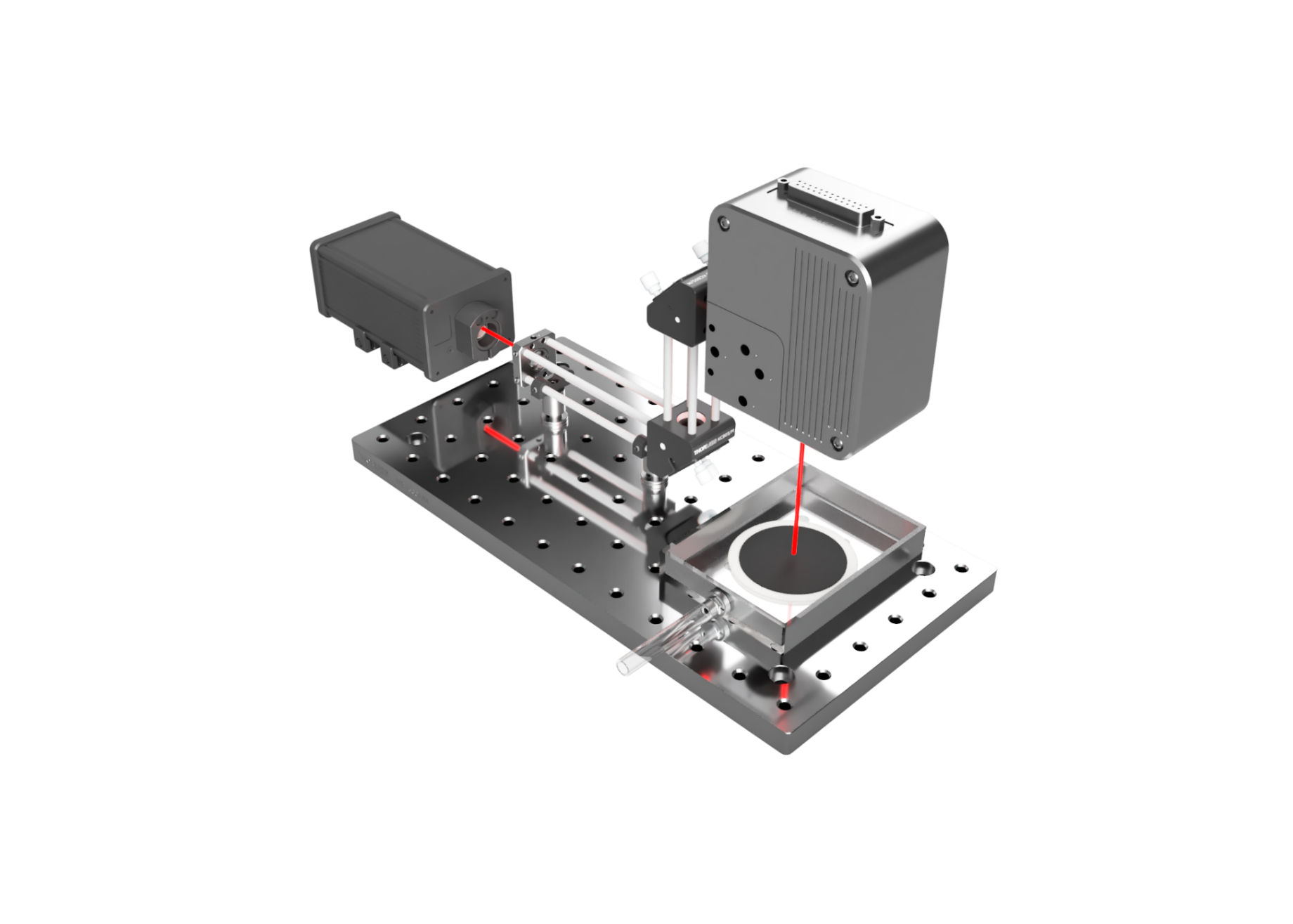
**

**Figure S1** Schematic illustration of the fabrication of LrEGO and M-LrEGO films. The laser beam was scanned across the EGO film. Constant Ar gas flow was applied on top of the EGO film.


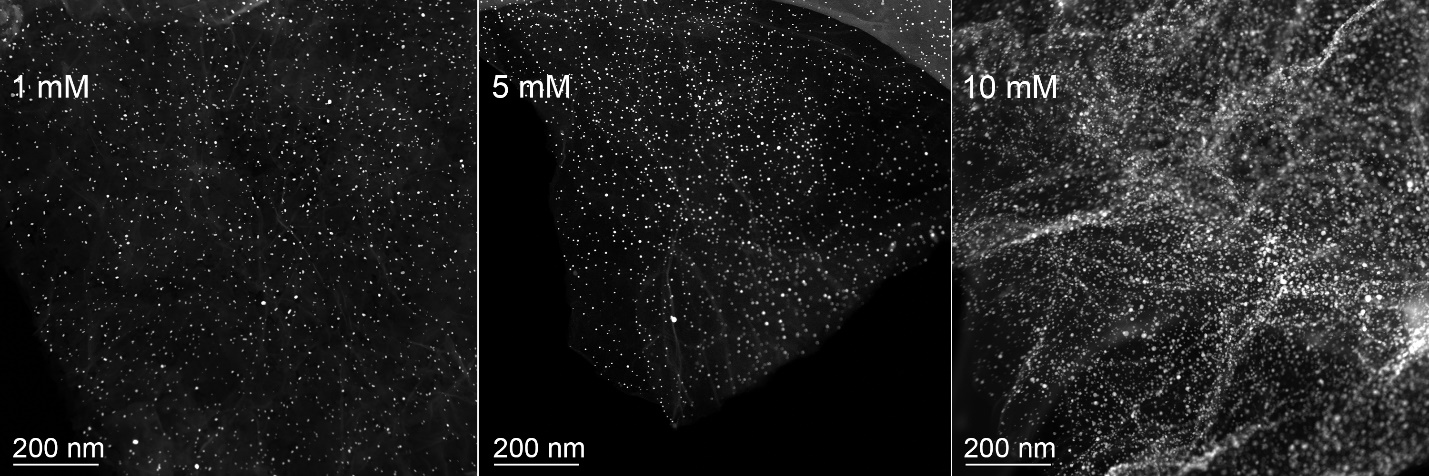


**(c)**

**(b)**

**(a)**

**Figure S2** STEM images of the Pt-LrEGO synthesized via 1064 nm laser irradiation at a laser fluence of 7.66 mJ cm^-2^ on PtCl_4_ filtered EGO films with a concetration of (**a**) 1, (**b**) 5 and (**c**) 10 mM, respectively.


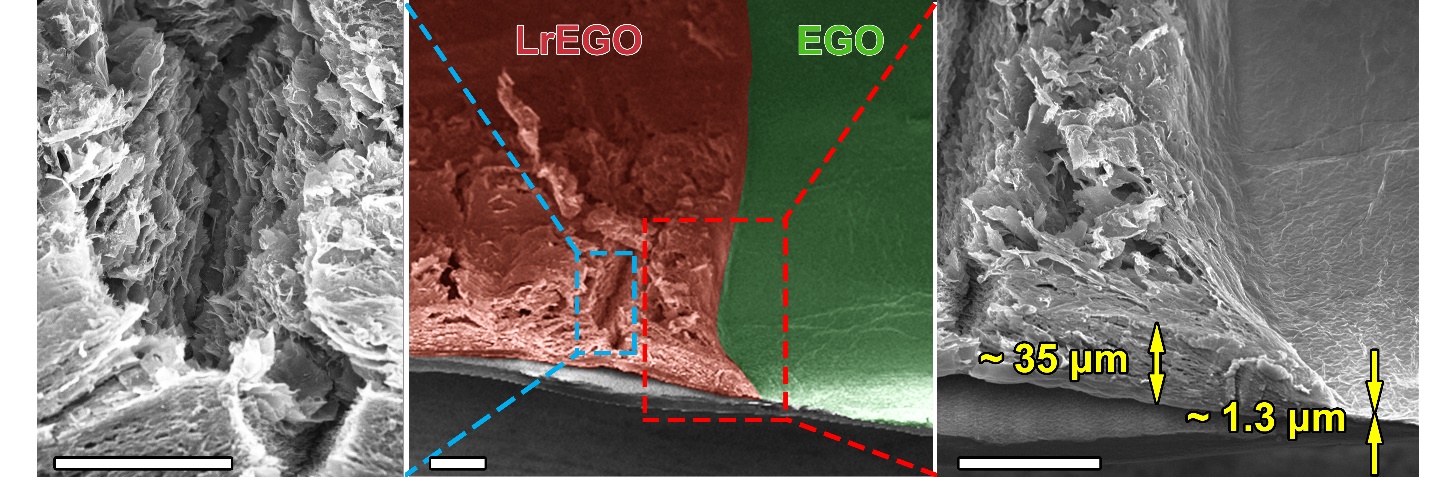


**(c)**

**(b)**

**(a)**

**Figure S3** FEG-SEM images of (**a**) and (**b**) representative LrGO revealing the reduction of LrGO and (**c**) the interface of GO and LrGO after 1064 nm laser irradiation at a laser fluence of 7.66 mJ cm^-2^.

**



**

**(b)**

**(a)**

**Figure S4** XPS high resolution C1s spectra of (**a**) pristine EGO and (**b**) LrEGO films.

**
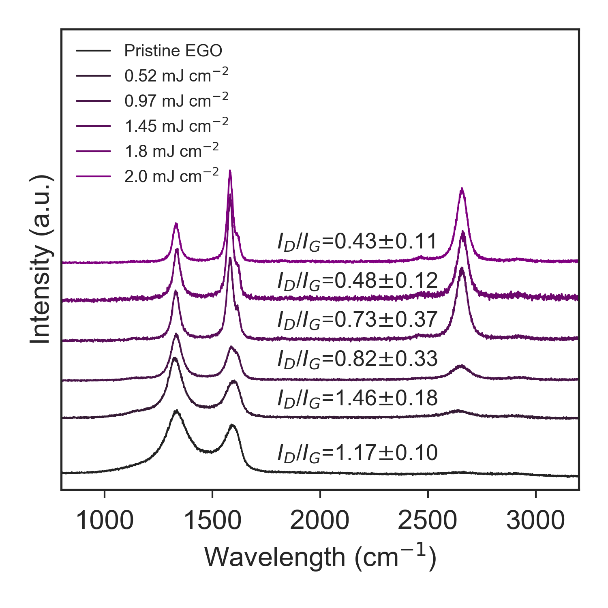

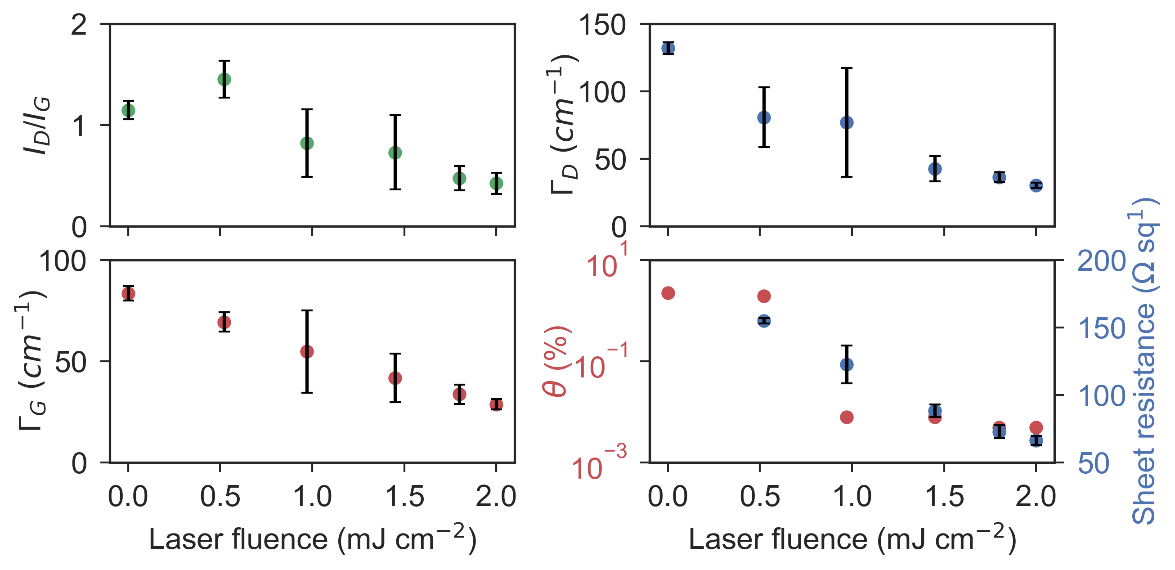
**

**(a)**

**(e)**

**(c)**

**(d)**

**(b)**

**Figure S5** (**a**) Raman spectra of the pristine EGO, and 355 nm laser treated samples at various laser fluences. The evolution of (**b**) *I*_D_*/I*_G_ ratio, (**c**) and (**d**) FWHM values of D and G bands, and (**e**) defect density and sheet resistance of EGO and LrEGO films.

**
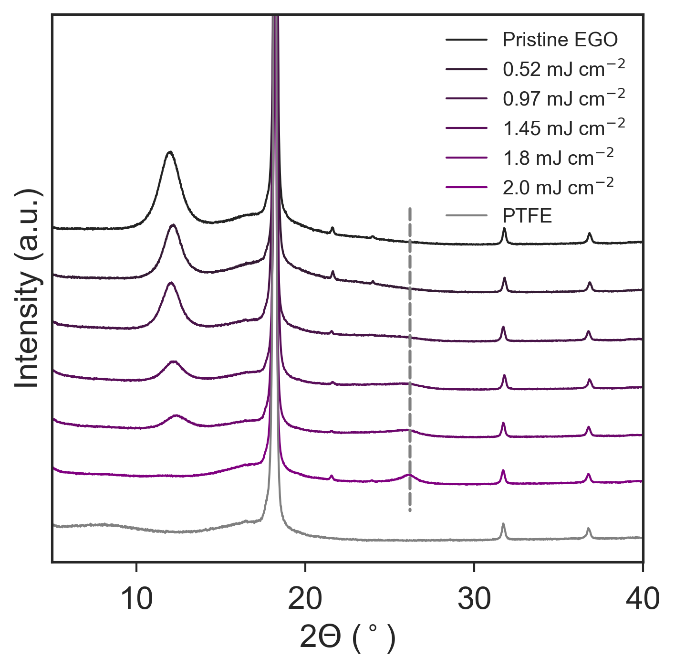
**

**Figure S6** XRD patterns of the pristine EGO and LrEGO films after 1064 nm laser irradiation at various laser fluences (the diffraction peaks centred at 2*θ* = 18.1°, 31.7° and 36.8° originated from the PTFE film).

**
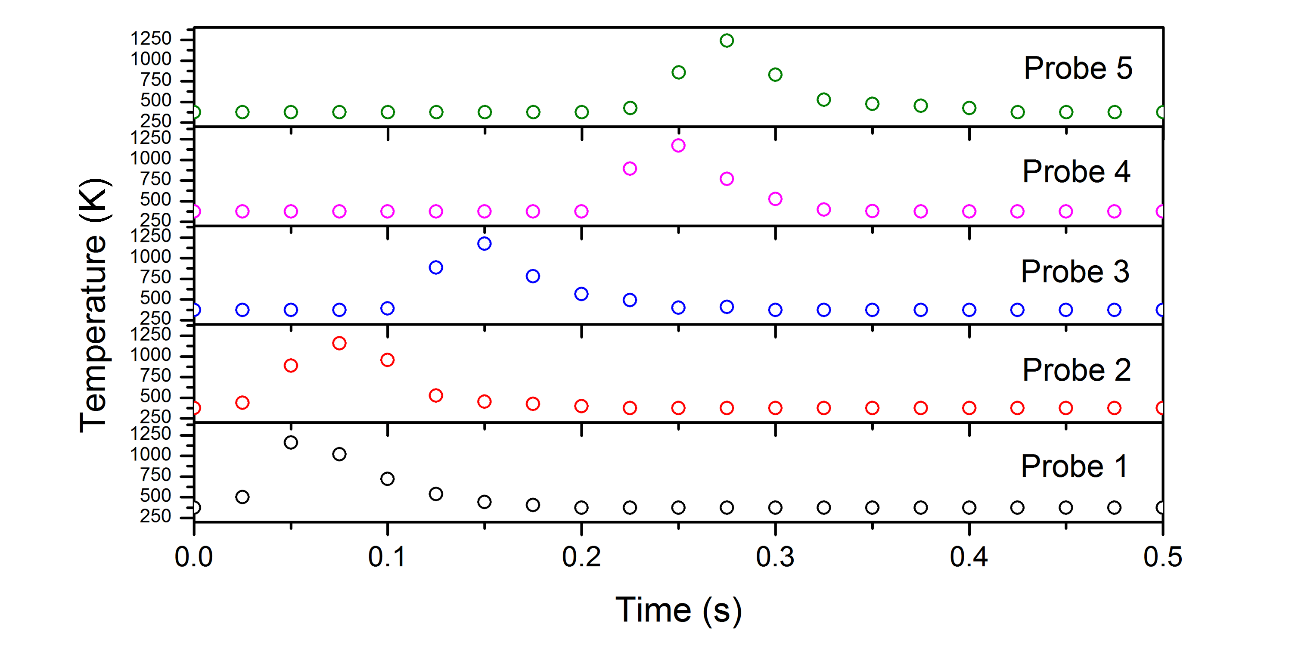
**

**Figure S7** Time trace of the surface temperature of 5 individual probe points across the patterned area, the temporal temperature profile presented in the main article was processed by averaging those individual probes dataset.


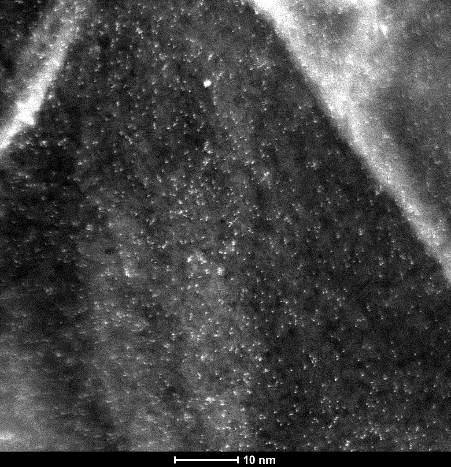

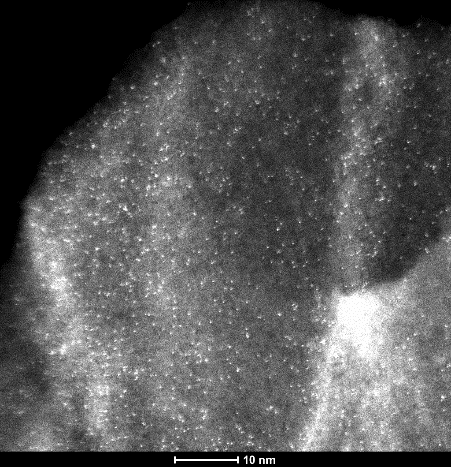

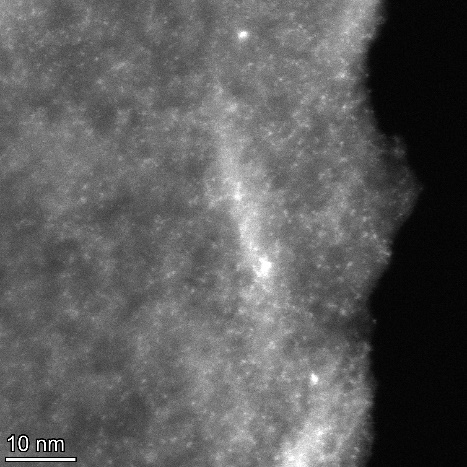


**(a)**

**(b)**

**(c)**

**Figure S8** Representative HR-STEM images of Pt5-LrEGO fabricated via 1064 nm laser irradiation at laser fluence of (**a**) 3.83, (**b**) 7.66 and (**c**) 11.49 mJ cm^-2^.


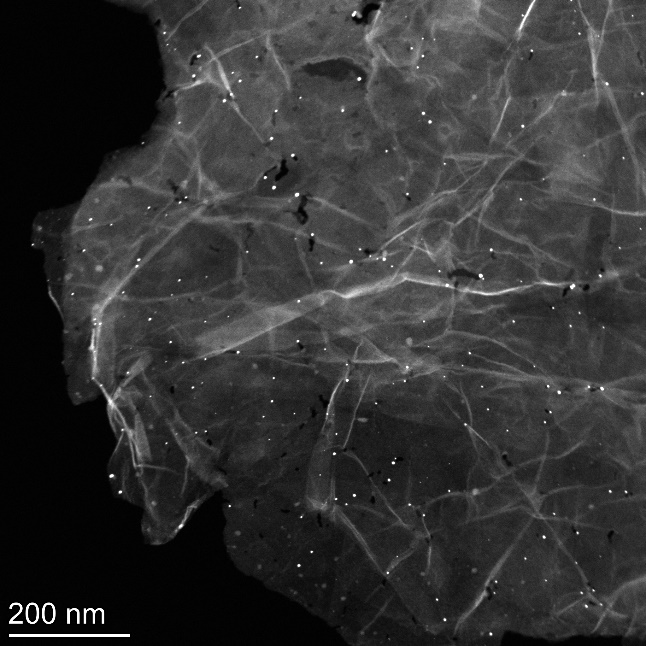

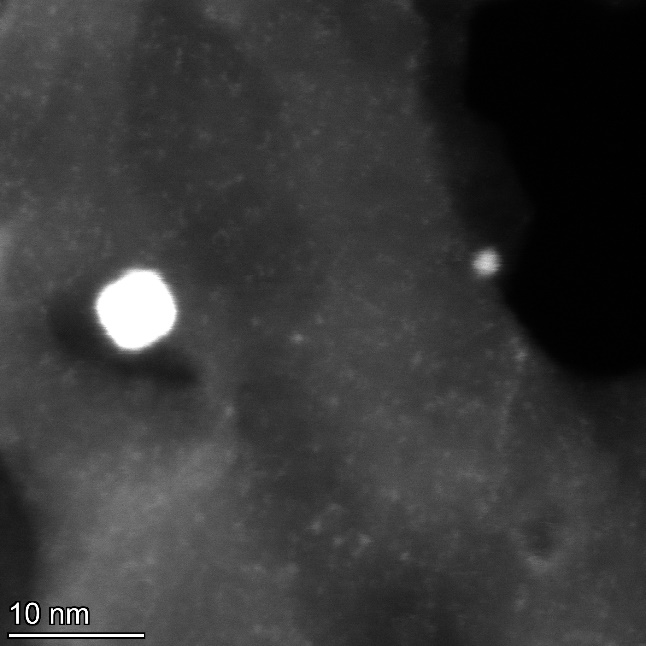


**(a)**

**(b)**

**Figure S9** Representative (**a**) STEM and (**b**) HR-STEM images of Pt5-LrEGO synthesised via 1064 nm laser irradiation at 7.66 mJ cm^-2^, with a scanning speed of 800 mm s^-1^. The co-existence of Pt nanoparticles and single atoms were clearly presented.


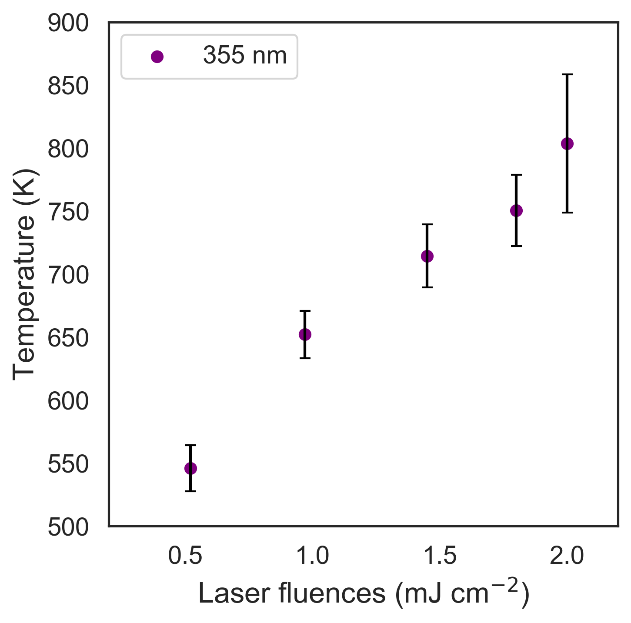


**Figure S10** The change of temperatures with the variation of laser fluences under 355 nm laser irradiation at a scanning speed of 1000 mm s^-1^.


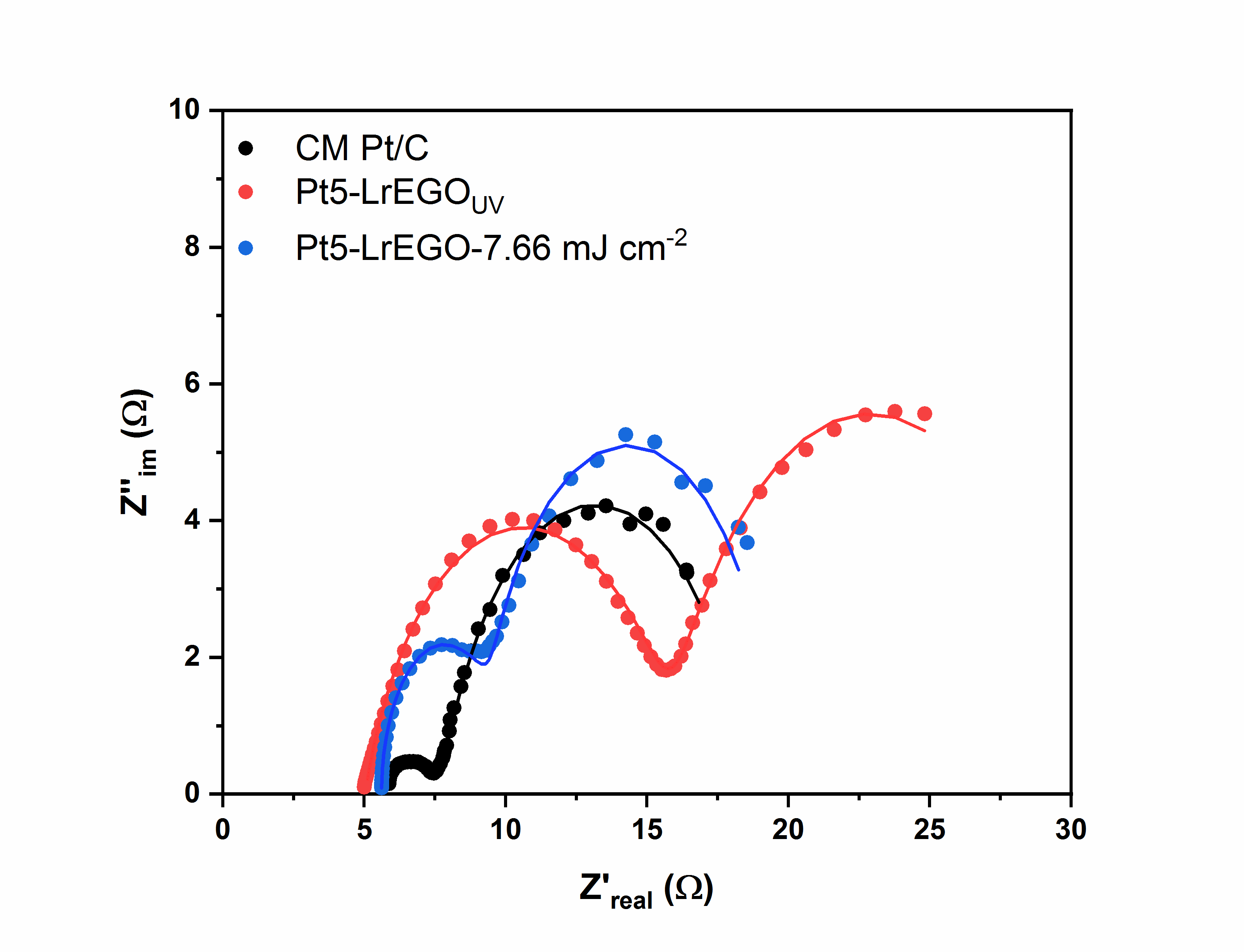

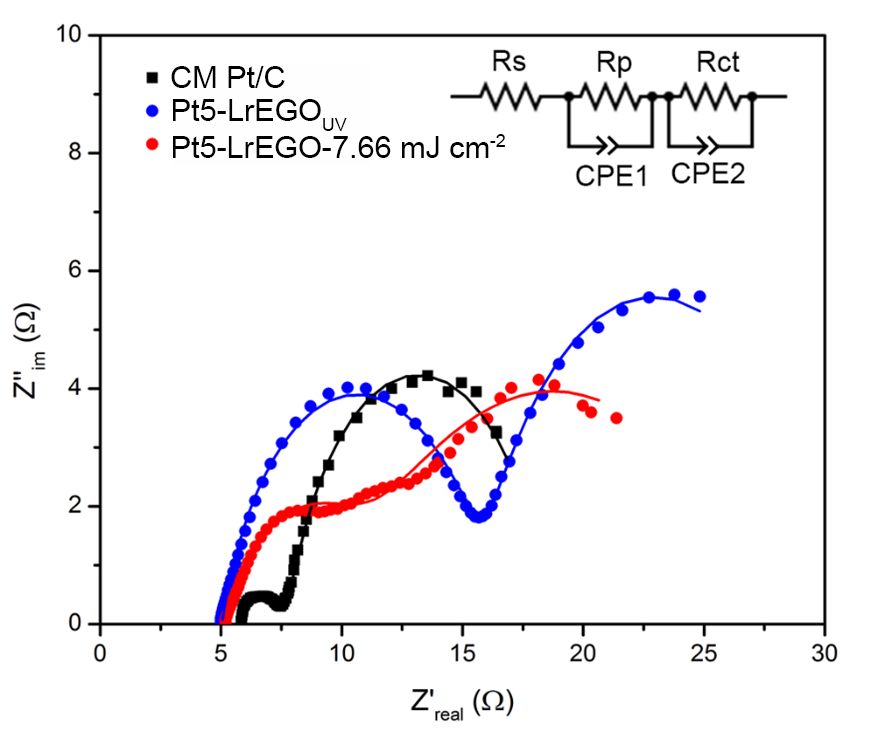


**Figure S11** The Nyquist plots of Pt5-LrEGO fabricated via 1064 nm laser irradiation at 7.66 mJ cm^-2^, Pt5-LrEGO_UV_ treated through 355 nm laser at 2.00 mJ cm^-2^ and the commercial Pt/C at an overpotential of 30 mV with 10 mV sinusoidal perturbations from 0.01 to 10^6^ Hz. The inset shows the equivalent circuit models, where *R*_s_ represents the solution resistance*, R*_p_ represents the surface absorption resistance and *R*_ct_ is the charge transfer resistance. The *R*_ct_ of CM Pt/C, Pt5-LrEGOUV and Pt5-LrEGO-7.66 mJ cm^-2^ were estimated to be 2.67, 9.88 and 14.23 Ω, respectively.

**Figure S12** Representative HAADF-STEM images of (**a and b**) Pt5-LrEGO-11.49, (**c and d**) Pt5-LrEGO-7.66, (**e and f**) Pt5-LrEGO-3.83 and (**g and h**) Pt5-LrEGO_UV_ after 10 hours chronopotentiometry test at a current density of 10 mA cm^-2^ at a rotating speed of 1600 rpm in N_2_-saturated 0.5 M H_2_SO_4_ electrolyte.


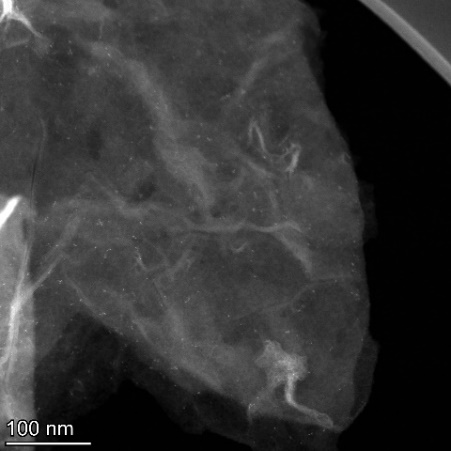

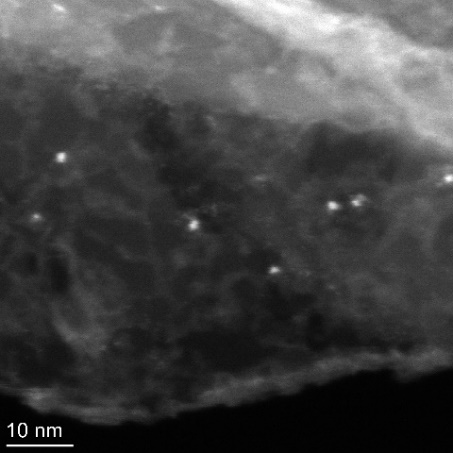

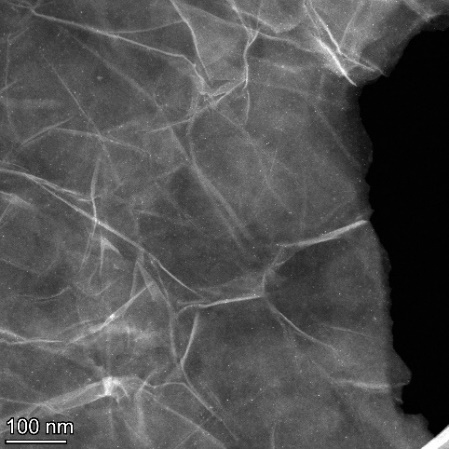

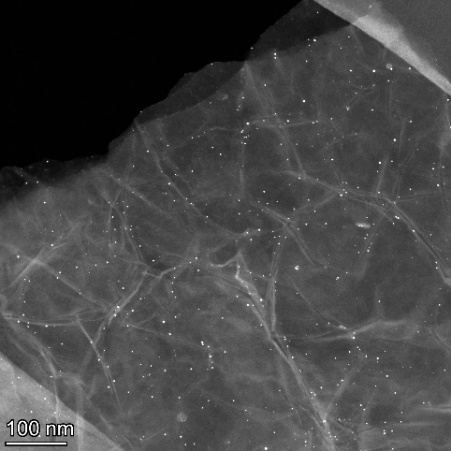

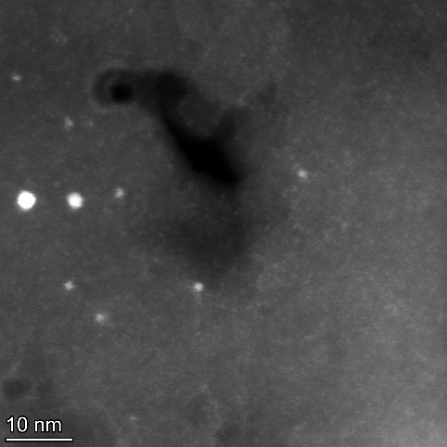

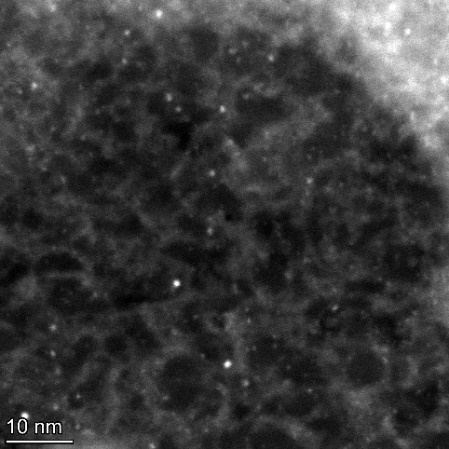

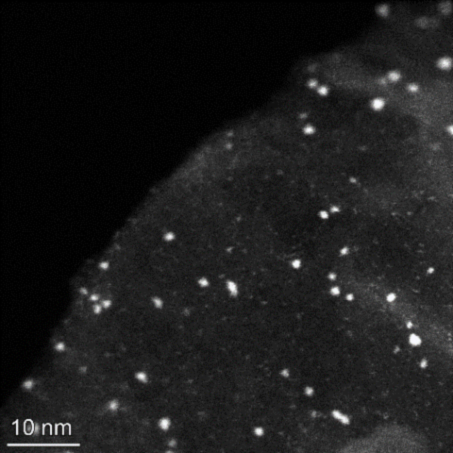

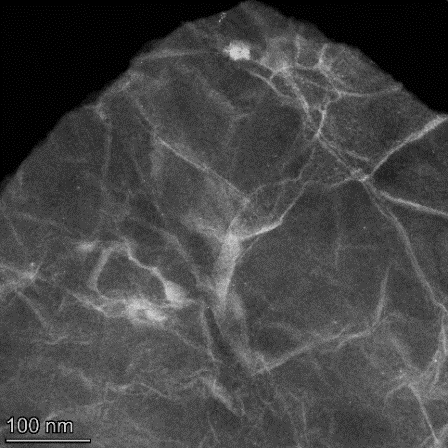


**(a)**

**(b)**

**(c)**

**(d)**

**(e)**

**(f)**

**(g)**

**(h)**

# Supporting tables

**Table S1** Summary of XPS high resolution Pt 4f spectra of EGO after PtCl_4_ and H_2_PtCl_6_ sorption.

| Component | Pt(IV) | | Pt(II) | | PtO/Pt(OH)_2_ | |
| --- | --- | --- | --- | --- | --- | --- |
| Assignment | PtCl_4_/H_2_PtCl_6_ | | PtCl_2_ | | PtO/Pt(OH)_2_ | |
|  | 4f5 | 4f7 | 4f5 | 4f7 | 4f5 | 4f7 |
| EGO-PtCl_4_ | 78.8 | 75.5 | 77.1 | 73.8 | 76.1 | 72.8 |
| EGO-H_2_PtCl_6_ | 78.8 | 75.5 | 77.2 | 73.9 | 76.0 | 72.6 |

**Table S2** Summary of XPS characterization of pristine EGO and LrEGO.

| Sample | Oxygen content (at.%) | Carbon content (at.%) | C 1s components (at.%) | | | | |
| --- | --- | --- | --- | --- | --- | --- | --- |
|  |  |  | C1s  284.5 eV | C−O  286.4 eV | C=O  287.9 eV | −COO−  289.2 eV | π to π*  290.8 eV |
| Pristine EGO | 20.04 | 79.96 | 57.88 | 39.94 | 1.53 | 0.98 | 0 |
| LrGO | 5.08 | 94.92 | 90.90 | 8.89 | 3.56 | 0 | 6.70 |

**Table S3** Summary of statistical Raman analysis of pristine EGO and EGO after 1064 nm laser irradiation at various laser fluences.

| Laser fluence (mJ cm^−2^) | Γ_D_ (cm^−1^) | Γ_G_ (cm^−1^) | *I*_D_*/I*_G_ | *L*_D_ (nm) | *θ* (%) |
| --- | --- | --- | --- | --- | --- |
| 0 | 132.12±4.32 | 83.61±3.58 | 1.17±0.10 | 1.16 | 2.25 |
| 3.83 | 129.56±11.20 | 77.72±6.04 | 1.12±0.02 | 1.14 | 2.36 |
| 5.11 | 78.27±15.24 | 58.98±8.24 | 1.31±0.19 | 1.18 | 2.20 |
| 6.38 | 46.38±10.52 | 54.70±4.76 | 1.26±0.07 | 14.81 | 0.014 |
| 7.66 | 39.50±8.43 | 43.64±9.39 | 0.97±0.27 | 16.92 | 0.010 |
| 8.93 | 36.36±4.59 | 35.56±4.38 | 0.63±0.19 | 21.21 | 0.006 |
| 10.21 | 31.89±2.25 | 32.09±3.64 | 0.56±0.19 | 22.53 | 0.006 |
| 11.49 | 29.34±3.94 | 27.70±1.34 | 0.35±0.11 | 28.70 | 0.004 |
| 12.76 | 30.13±2.13 | 29.60±2.45 | 0.47±0.08 | 24.65 | 0.005 |

| Laser fluence (mJ cm^−2^) | Γ_D_ (cm^−1^) | Γ_G_ (cm^−1^) | *I*_D_*/I*_G_ | *θ* (%) |
| --- | --- | --- | --- | --- |
| 0 | 132.12±4.32 | 83.61±3.58 | 1.17±0.10 | 2.25 |
| 0.52 | 80.86±22.25 | 69.45±4.81 | 1.46±0.18 | 1.96 |
| 0.97 | 77.02±40.27 | 54.90±20.32 | 0.82±0.33 | 0.008 |
| 1.45 | 42.68±9.29 | 41.88±11.96 | 0.73±0.37 | 0.008 |
| 1.80 | 36.36±3.57 | 33.75±4.79 | 0.48±0.12 | 0.005 |
| 2.00 | 30.37±1.89 | 28.78±2.61 | 0.43±0.11 | 0.005 |

**Table S4** Summary of statistical Raman analysis of pristine EGO and EGO after 355 nm laser irradiation at various laser fluences.

**Table S5** Material properties of the simulated EGO under IR laser irradiation.

| Material Properties | EGO | Units | Ref. |
| --- | --- | --- | --- |
| Density | 2200 | kg m^-3^ | ^5,6^ |
| Heat capacitance | 2000 | J kg^-1^K^-1^ | ^7,8^ |
| In-plane thermal conductivity | 8.8 | W m^-1^K^-1^ | ^9-11^ |
| Cross-plane thermal conductivity | 0.09 | W m^-1^K^-1^ | ^11^ |
| Absorptivity @ 1064 nm | 0.6 |  | ^12,13^ |

**Table S6** HER experimental conditions and activities of Pt5-rEGO, CM Pt/C and other reported platinum-based SACs tested in 0.5 M H_2_SO_4_ electrolyte.

| Catalysts | Electrode | Electrolyte | Catalyst loading^b)^ (mg cm^−2^) | *η*^c)^ @ 10mA cm^−2^ (mV) | Tafel slope (mV dec^−1^) | Pt mass activity (mA μg_Pt_^-1^) | Ref. |
| --- | --- | --- | --- | --- | --- | --- | --- |
| Pt5-LrEGO | GCE^a)^ | 0.5 M H_2_SO_4_ | ~0.255 | 42.3 | 33.8 | 12.36 @ *η*_50 mV_ | This work |
| CM Pt/C (20 wt.%) |  |  |  | 32.2 | 32.3 | 0.83 @ *η*_50 mV_ |  |
| Pt/np-Co_0.85_Se | Pt/np-Co_0.85_Se | 0.5 M H_2_SO_4_ | N/A | 58 | 26 | 13.57 @ *η*_50 mV_ | ^14^ |
| Pt_1_/OLC | GCE | 0.5 M H_2_SO_4_ | 0.51 | ~38 | 36 | ~13.46 @ *η*_50 mV_ | ^15^ |
| Pt_1_/graphene |  |  |  | ~56.6 | 42 | ~4.93 @ *η*_50 mV_ |  |
| Pt SA/m-WO_3-x_ | GCE | 0.5 M H_2_SO_4_ | ~0.204 | 47 | 45 | 12.8 @ *η*_50 mV_ | ^16^ |
| ALD50Pt/NGNs | GCE | 0.5 M H_2_SO_4_ | ~0.077 | ~41 | 29 | 10.1 @ *η*_50 mV_ | ^17^ |
| Pt_1_/NMC | GCE | 0.5 M H_2_SO_4_ | ~0.204 | 29 | 26 | 10 @ *η*_50 mV_ | ^18^ |
| Pt-GDY2 | Ti foil | 0.5 M H_2_SO_4_ | 4.65 (μg_Pt_ cm^-2^) | N/A | N/A | ~10 @ *η*_50 mV_ | ^19^ |
| Pt single atoms | GCE | 0.5 M H_2_SO_4_ | ~0.153 | ~39 | 33.2 | 10 @ *η*_50 mV_ | ^20^ |
| Mo_2_TiC_2_T_x_-Pt_SA_ | Carbon paper | 0.5 M H_2_SO_4_ | 1 | ~32.8 | 30 | 8.3 @ *η*_77 mV_ | ^21^ |
| Pt@PCM | GCE | 0.5 M H_2_SO_4_ | ~0.107 | 105 | 65.3 | ~5.13 @ *η*_50 mV_ | ^22^ |
| Pt-MoO_2_/MWCNT_S_ | GCE | 0.5 M H_2_SO_4_ | 0.47 | 60 | 43 | ~2.77 @ *η_50_* _mV_ | ^23^ |
| Pt_1_@Fe-N-C | GCE | 0.5 M H_2_SO_4_ | ~0.3 | 60 | 42 | ~0.9 @ *η*_50 mV_ | ^24^ |
| Pt/def-WO_3_@CFC | GCE | 0.5 M H_2_SO_4_ | ~0.255 | 42 | 61 | ~0.8 @ *η*_50 mV_ | ^25^ |

^a)^ Glassy carbon electrode. ^b)^ The catalyst loading, if not specific noted, denotes the total mass of catalysts loaded on the working electrode, and  ^c)^ Overpotential.

# References

1 Cao, J. Y. *et al.* Two-Step Electrochemical Intercalation and Oxidation of Graphite for the Mass Production of Graphene Oxide. *Journal of the American Chemical Society* **139**, 17446-17456, doi:10.1021/jacs.7b08515 (2017).

2 Xiao, L. *et al.* Fast Adaptive Thermal Camouflage Based on Flexible VO_2_/Graphene/CNT Thin Films. *Nano Letters* **15**, 8365-8370, doi:10.1021/acs.nanolett.5b04090 (2015).

3 Zhou, Y. *et al.* Microstructuring of Graphene Oxide Nanosheets Using Direct Laser Writing. *Advanced Materials* **22**, 67-71, doi:10.1002/adma.200901942 (2010).

4 Chen, X., Chen, Y., Yan, M. & Qiu, M. Nanosecond Photothermal Effects in Plasmonic Nanostructures. *ACS Nano* **6**, 2550-2557, doi:10.1021/nn2050032 (2012).

5 Wicklein, B. *et al.* Thermally insulating and fire-retardant lightweight anisotropic foams based on nanocellulose and graphene oxide. *Natural Nanotechnology* **10**, 277-283, doi:10.1038/nnano.2014.248 (2015).

6 Stankovich, S. *et al.* Synthesis of graphene-based nanosheets via chemical reduction of exfoliated graphite oxide. *Carbon* **45**, 1558-1565, doi:[10.1016/j.carbon.2007.02.034](https://doi.org/10.1016/j.carbon.2007.02.034) (2007).

7 McAllister, M. J. *et al.* Single Sheet Functionalized Graphene by Oxidation and Thermal Expansion of Graphite. *Chemistry of Materials* **19**, 4396-4404, doi:10.1021/cm0630800 (2007).

8 Butland, A. T. D. & Maddison, R. J. The specific heat of graphite: An evaluation of measurements. *Journal of Nuclear Materials* **49**, 45-56, doi:[10.1016/0022-3115(73)90060-3](https://doi.org/10.1016/0022-3115(73)90060-3) (1973).

9 Mu, X., Wu, X., Zhang, T., Go, D. B. & Luo, T. Thermal Transport in Graphene Oxide – From Ballistic Extreme to Amorphous Limit. *Scientific Reports* **4**, 3909, doi:10.1038/srep03909 (2014).

10 Morath, C. J. *et al.* Picosecond optical studies of amorphous diamond and diamondlike carbon: Thermal conductivity and longitudinal sound velocity. *Journal of Applied Physics* **76**, 2636-2640, doi:10.1063/1.357560 (1994).

11 Renteria, J. D. *et al.* Strongly Anisotropic Thermal Conductivity of Free-Standing Reduced Graphene Oxide Films Annealed at High Temperature. *Advanced Functional Materials* **25**, 4664-4672, doi:10.1002/adfm.201501429 (2015).

12 Yang, H. *et al.* Highly conductive free-standing reduced graphene oxide thin films for fast photoelectric devices. *Carbon* **115**, 561-570, doi:[10.1016/j.carbon.2017.01.047](https://doi.org/10.1016/j.carbon.2017.01.047) (2017).

13 Tian, H., Cao, Y., Sun, J. & He, J. Enhanced broadband photoresponse of substrate-free reduced graphene oxide photodetectors. *RSC Advances* **7**, 46536-46544, doi:10.1039/C7RA09826J (2017).

14 Jiang, K. *et al.* Single platinum atoms embedded in nanoporous cobalt selenide as electrocatalyst for accelerating hydrogen evolution reaction. *Nature Communications* **10**, 1743, doi:10.1038/s41467-019-09765-y (2019).

15 Liu, D. *et al.* Atomically dispersed platinum supported on curved carbon supports for efficient electrocatalytic hydrogen evolution. *Nature Energy* **4**, 512-518, doi:10.1038/s41560-019-0402-6 (2019).

16 Park, J. *et al.* Investigation of the Support Effect in Atomically Dispersed Pt on WO3−x for Utilization of Pt in the Hydrogen Evolution Reaction. *Angewandte Chemie International Edition* **58**, 16038-16042, doi:10.1002/anie.201908122 (2019).

17 Cheng, N. C. *et al.* Platinum single-atom and cluster catalysis of the hydrogen evolution reaction. *Nature Communications* **7**, 13638, doi:10.1038/ncomms13638 (2016).

18 Wei, H. H. *et al.* Ultralow-temperature photochemical synthesis of atomically dispersed Pt catalysts for the hydrogen evolution reaction. *Chemical Science* **10**, 2830-2836, doi:10.1039/c8sc04986f (2019).

19 Yin, X.-P. *et al.* Engineering the Coordination Environment of Single-Atom Platinum Anchored on Graphdiyne for Optimizing Electrocatalytic Hydrogen Evolution. *Angewandte Chemie International Edition* **57**, 9382-9386, doi:10.1002/anie.201804817 (2018).

20 Zhang, L. *et al.* Atomic layer deposited Pt-Ru dual-metal dimers and identifying their active sites for hydrogen evolution reaction. *Nature Communications* **10**, 4936, doi:10.1038/s41467-019-12887-y (2019).

21 Zhang, J. *et al.* Single platinum atoms immobilized on an MXene as an efficient catalyst for the hydrogen evolution reaction. *Nature Catalysis* **1**, 985-992, doi:10.1038/s41929-018-0195-1 (2018).

22 Zhang, H. *et al.* Dynamic traction of lattice-confined platinum atoms into mesoporous carbon matrix for hydrogen evolution reaction. *Science Advances* **4**, eaao6657, doi:10.1126/sciadv.aao6657 (2018).

23 Xie, X. *et al.* Ultralow Pt Loaded Molybdenum Dioxide/Carbon Nanotubes for Highly Efficient and Durable Hydrogen Evolution Reaction. *The Journal of Physical Chemistry C* **121**, 24979-24986, doi:10.1021/acs.jpcc.7b08283 (2017).

24 Zeng, X. J. *et al.* Single-Atom to Single-Atom Grafting of Pt_1_ onto Fe-N_4_ Center: Pt_1_@Fe-N-C Multifunctional Electrocatalyst with Significantly Enhanced Properties. *Advanced Energy Materials* **8**, 1701345, doi:10.1002/aenm.201701345 (2018).

25 Tian, H. *et al.* Oxygen vacancy-assisted hydrogen evolution reaction of the Pt/WO_3_ electrocatalyst. *Jouranl of Materials Chemistry A* **7**, 6285-6293, doi:10.1039/C8TA12219A (2019).
